# Multifunctional fucoidan-loaded Zn-MOF-encapsulated microneedles for MRSA-infected wound healing

**DOI:** 10.1186/s12951-024-02398-4

**Published:** 2024-04-04

**Authors:** Zichao Jiang, Jingyi Li, Jiahao Wang, Yixiao Pan, Shuailong Liang, Yihe Hu, Long Wang

**Affiliations:** 1 Department of Orthopedics, Xiangya Hospital, Central South University, Changsha, China; 2 Department of Orthopedics, First Affiliated Hospital, School of Medicine, Zhejiang, China; 3 University Hunan Engineering Research Center of Biomedical Metal and Ceramic Implants, Xiangya Hospital, Central South University, Changsha, China; 4 National Clinical Research Center for Geriatric Disorders, Xiangya Hospital, Central South University, Changsha, China; 5 Hunan Key Laboratory of Aging Biology, Xiangya Hospital, Central South University, Changsha, China

**Keywords:** Fucoidan, Microneedle, Subcelluar targeting, Transdermal delivery, Infected wound

## Abstract

Infected wound healing remains a challenging task in clinical practice due to several factors: (I) drug-resistant infections caused by various pathogens, (II) persistent inflammation that hinders tissue regeneration and (III) the ability of pathogens to persist intracellularly and evade antibiotic treatment. Microneedle patches (MNs), recognized for their effecacious and painless subcutaneous drug delivery, could greatly enhance wound healing if integrated with antibacterial functionality and tissue regenerative potential. A multifunctional agent with subcellular targeting capability and contained novel antibacterial components, upon loading onto MNs, could yield excellent therapeutic effects on wound infections. In this study, we sythesised a zeolitic imidazolate framework-8 nanoparticles (ZIF-8 NPs) loaded with low molecular weight fucoidan (Fu) and further coating by hyaluronic acid (HA), obtained a multifunctional HAZ@Fu NPs, which could hinders Methicillin-resistant *Staphylococcus aureus* (MRSA) growth and promotes M2 polarization in macrophages. We mixed HAZ@Fu NPs with photocrosslinked gelatin methacryloyl (GelMA) and loaded it into the tips of the MNs (HAZ@Fu MNs), administered to mice model with MRSA-infected full-thickness cutaneous wounds. MNs are able to penetrate the skin barrier, delivering HAZ@Fu NPs into the dermal layer. Since cells within infected tissues extensively express the HA receptor CD44, we also confirmed the HA endows the nanoparticles with the ability to target MRSA in subcellular level. In vitro and in vivo murine studies have demonstrated that MNs are capable of delivering HAZ@Fu NPs deep into the dermal layers. And facilitated by the HA coating, HAZ@Fu NPs could target MRSA surviving at the subcellular level. The effective components, such as zinc ions, Fu, and hyaluronic acid could sustainably released, which contributes to antibacterial activity, mitigates inflammation, promotes epithelial regeneration and fosters neovascularization. Through the RNA sequencing of macrophages post co-culture with HAZ@Fu, the Kyoto Encyclopedia of Genes and Genomes (KEGG) pathway analysis reveals that the biological functionalities associated with wound healing could potentially be facilitated through the PI3K-Akt pathway. The results indicate that the synergistic application of HAZ@Fu NPs with biodegradable MNs may serve as a significant adjunct in the treatment of infected wounds. The intricate mechanisms driving its biological effects merit further investigation.

**Supplementary Information:**

The online version contains supplementary material available at 10.1186/s12951-024-02398-4.

## Introduction

Skin wounds, commonly caused by daily trauma, created a disruption in the primary antibacterial barrier, make it becomes vulnerable to infection by various microorganisms. It can ultimately lead to the development of a chronically infected wound which result in severe pain and delayed healing. Additionally, wounds that fail to heal promptly may become prone to further infections and persistent inflammation [1, 2]. Given the dosage limitations and potential systemic toxicity of antibiotics, the healing of infected wounds has garnered increasing attention in recent research [3–5].

Among various wound infections, *Staphylococcus aureus* (*S. aureus*) is responsible for approximately 80% of all skin and soft tissue infections. And more than 30% of patients with *S. aureus* infection develop chronic or recurring infections within three months, even after receiving antibacterial treatment [6, 7]. Among the various factors contributing to this issue, the capacity of *S. aureus* to survive within host cells is widely acknowledged as a key determinant in the persistence and recurrence of these infections [8, 9]. Increasing evidence has shown that *S. aureus* can resist the fusion of phagosomes with lysosomes, thereby multiplying within the phagolysosomes of macrophages and also to persist in human keratinocytes [10, 11]. This characteristic allows *S. aureus* to evade both the immune system and antibiotics, with the potential to regrow when the environment permits [12]. The development of antimicrobial resistance in these internalized colonies can be caused by factors such as intraphagolysosomal concentrations of antimicrobials lower than the minimum inhibitory concentration (MIC), the acidic pH of phagolysosomes can influence antibiotic activity and various antimicrobials tend to accumulate in subcellular structures other than lysosomes [13, 14].

MRSA, as a strain of *S. aureus* that is resistant to multiple drugs, is a substantial threat to human health. In the clinical setting, glycopeptide antibiotics epitomized by vancomycin (Van) are typically employed to combat MRSA infections. Addressing the challenge of MRSA infected wound, we suppose to develop a composite material that combines new antimicrobial agents with the ability to targeting intracellular MRSA.

In our previous study, we observed that low molecular weight Fu exhibits significant effectiveness in suppressing MRSA growth [15]. Fu is a class of fucose-rich sulfated polysaccharides derived from brown algae, known for its various biological benefits such as antiviral, antitumor, and anti-inflammatory effects [16–18]. The biological function of Fu is greatly influenced by its molecular weight [19, 20], the depolymerized form of fucoidan has been proven to effectively inhibit the growth and proliferation of various bacteria, including *Escherichia coli*, *S. aureus*, and *Helicobacter pylori*, thereby demonstrating its broad-spectrum antibacterial potential with extremely low toxicity or side effects [21–23]. This suggests that Fu could be a promising candidate in the continuous search for effective antimicrobial agents.

Recognizing the antimicrobial potential of metal ions, our study highlights the possibility of leveraging metal-organic framework (MOF) materials for the creation of innovative antibacterial agents. MOF is a unique class of nanoporous materials formed from metal ions interconnected by organic ligands via coordinate covalent bonds, are emerging as promising nanoplatforms for precision drug delivery owing to their unique structural properties [24, 25]. Among them, zeolitic imidazolate framework-8 (ZIF-8) exhibits commendable antibacterial properties, characteristics conducive to surface modification, high biocompatibility, adjustable drug release characteristics, and substantial drug loading capacity, has become a popular choice for design of drug-loaded NPs in recent investigation [26, 27]. The discharge of zinc ions from ZIF-8 disrupts the bacterial membrane integrity and triggers the generation of reactive oxygen species, leading to the elimination of the bacteria [28, 29]. Additionally, zinc ions have been shown to enhance the expression of anti-inflammatory cytokines, thereby promoting an anti-inflammatory environment [28]. Considering these properties of ZIF-8, we propose utilizing it to encapsulate Fu (ZIF@Fu), which, after suitable modifications, could target to intracellular MRSA.

To enable ZIF@Fu to target subcellular structures, specifically lysosomes, we are incorporating hyaluronic acid (HA) into our design. HA is a naturally occurring substance in skin tissue, renowned for its extensive application in wound healing, skincare, and tumor targeting in recent years [29, 30]. Cluster of differentiation 44 (CD44), the prominent cell receptor for HA, plays a crucial role in HA binding and endocytosis. CD44 had been identified as the most widely distributed receptor in epithelial tissues where colonization and infection occur [31]. Commonly overexpressed in various cell types, especially some kinds of cancer cells [32], thus HA-modified materials are often used for targeted therapy and drug delivery. CD44 also overexpress in keratinocytes, fibroblasts [33], activated macrophages (M1 > M0 ≥ M2 polarization) [34]. Moreover, CD44 is known to facilitate the cellular uptake of HA, which is subsequently degraded within lysosomes [35]. Previous research had confirmed that HA-coated NPs can internalized by cells and subsequently delivered to lysosomes [36]. HA can directly substitute the organic linkers in ZIF-8, endows the NPs with targeting ability, also leveraging the beneficial wound-healing properties of HA.

For skin infection wounds, topical application is clearly more effective. Soluble MNs are a promising medical tool, allowing painless transdermal drug delivery through a minimally invasive method and have been extensively employed in various applications, including vaccinations, cancer treatments, skin therapies, and even in cosmetic products [37, 38]. The tip of MN is typically composed of therapeutic agents and dissolving polymers, the vast majority of microneedle lengths were between 500 and 1000 μm, typically averaging around 600 μm [39–41], which can creates temporary channels in the outer layer of the skin, thus allowing the delivery of different therapeutic compounds which otherwise would be incapable of delivery via the transdermal route [38, 42]. The tip height of the microneedle is sufficient to breach the stratum corneum yet does not extend deep enough to contact the subcutaneous nerve endings. All these characteristics confer MNs with great potential for wound treatment [43, 44].

It inspired us to design multifunctional MNs with degradability, antibacterial, and anti-inflammatory properties, specifically for enhancing the healing of infected wounds. MNs embedded with HAZ@Fu nanoparticles utilize a biocompatible gelatin methacryloyl (GelMA) hydrogel at their tips [45, 46]. The backing layer of the microneedle patch is made from dissolvable polyvinyl acetate (PVA), chosen particularly for its flexible nature, PVA not only bolsters the mechanical stability of the MN but also guarantees that the layer dissolves completely upon application [47].

In this study, we encapsulated HAZ@Fu into a photocrosslinked GelMA hydrogel, and synthesized MNs by using a molding method to form HAZ@Fu MN. Upon application to wounds infected by MRSA, these MNs enabled a sustained release of encapsulated HAZ@Fu NPs deep into the wound sites. Intriguingly, the HAZ@Fu NPs could specifically target MRSA internalized in the lysosomes at a subcellular level, releasing their constituent components. The zinc ions and Fu worked in concert to combat the MRSA, while also promoting M2 macrophage polarization, and releasing wound-friendly HA. By leveraging these properties, we demonstrated that HAZ@Fu MNs could exert a robust antibacterial effect against MRSA, also promote regeneration of epithelial and neovascularization. HAZ@Fu MN holds substantial promise for the treatment of infected wounds and show potential for broader clinical applications.

## Results and discussion

### Preparation and characterization of HAZ@Fu

The list of abbreviations relevant to this study is provided in Table. S1. Biocompatible and porous ZIF-8 was synthesized by a reaction between 2-methylimidazole (2-MIM) and Zn^2+^ salts in DI water. ZIF@Fu was prepared using a one-step synthesis method [27] and the Fu-loading ratio of HAZ@Fu and ZIF@Fu were detected by an indirect method [48]. Initially, Fu was mixed with Zn^2+^ salts, allowing the sulfonic acid groups in Fu to form coordination bonds with zinc ions. After further assembly with 2-MIM, the majority of Fu molecules were embedded into the framework, resulting in a high drug payload. The total weight of the ZIF, HAZ, ZIF@Fu and HAZ@Fu was measured after freeze-drying. The total weight of ZIF, HAZ, ZIF@Fu and HAZ@Fu NPs from 20 mL 2-MIM systems were 60, 68, 84 and 88 mg, respectively. Then the ZIF@Fu or HAZ@Fu was dissolved destroied by 10 mL EDTA (0.1 M), and the substance except for Fu was removed utilizing a semipermeable membrane with a 1000 Molecular Weight Cut-Off (MWCO). Then the remaining solution was then lyophilized and weighed to obtain the mass of the encapsulated Fu. The drug-loading efficiency was calculated according to the following formula: Drug loading (%) = W_drug_/W_total_ × 100%. The weight of Fu within ZIF@Fu or HAZ@Fu was approximately 13.52 mg, and the drug loading efficiency was calculated to be around 16.1%.

**Fig. 1:**
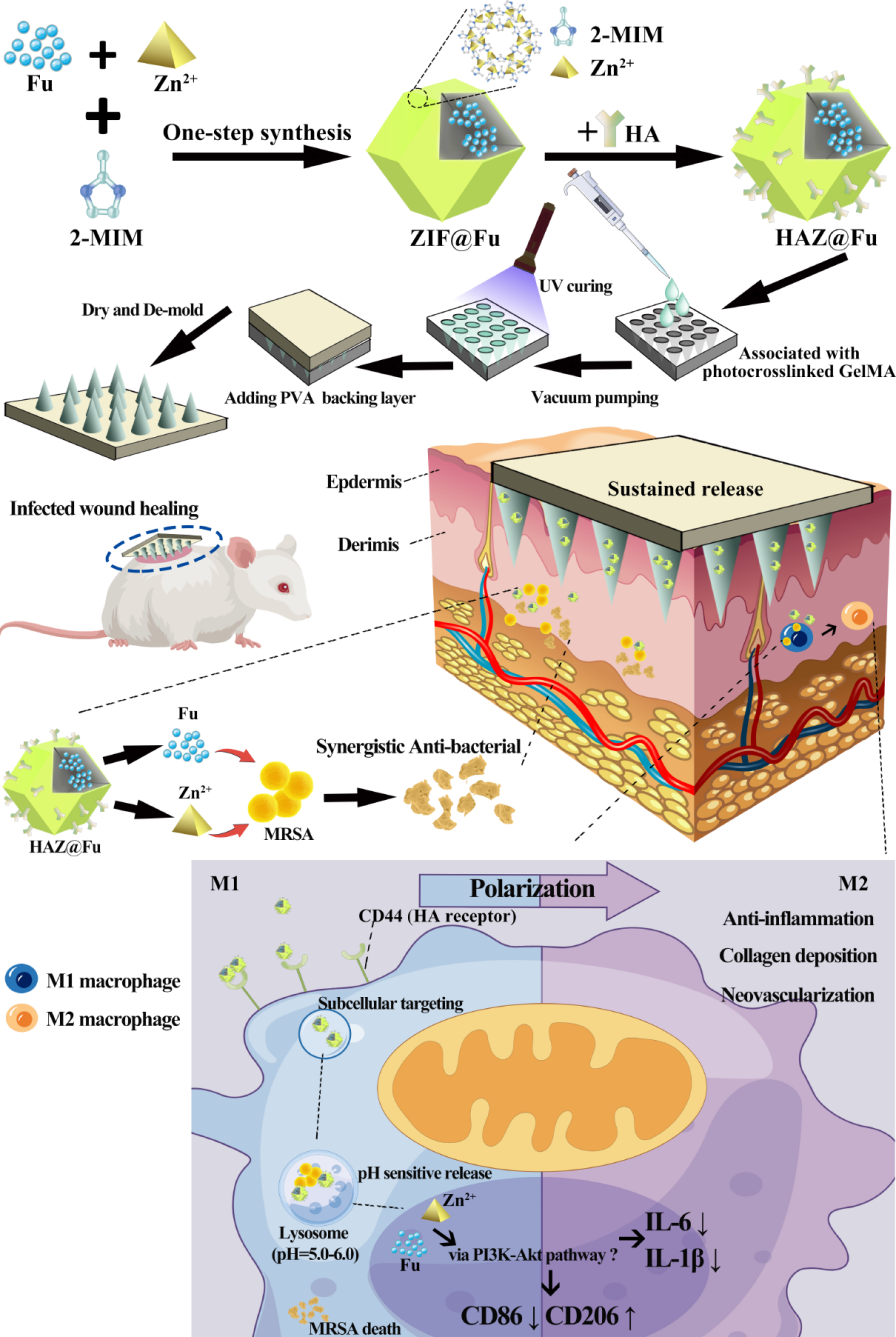
Schematic diagram of the preparation process of the HAZ@Fu MN via a template replication method, and its application encompassing both the process and mechanism of treatment for infected wounds

The morphology, particle size and surface potential of the NPs were analyzed by scanning electron microscope (SEM) and dynamic light scattering (DLS). As depicted in Fig. 1A, both ZIF-8 and ZIF@Fu NPs showed a uniform rhombic dodecahedron structure with sharp edges. In contrast, HAZ and HAZ@Fu exhibited smoother edges of the dodecahedron structure, which was likely attributable to the HA coating. DLS revealed that the hydrodynamic diameters of all the NPs were larger compared to those observed in the SEM images (Fig. 1C; Table 1). The average hydrodynamic diameter of ZIF-8 was 116.0 ± 0.7 nm. Both Fu loading and HA coating resulted in an increase in diameter, likely due to the additional material and layers incorporated into the nanoparticle structure. Consequently, the diameters of HAZ, ZIF@Fu, and HAZ@Fu NPs measured to be 168.5 ± 1.5, 222.5 ± 3.6, and 283.1 ± 8.4 nm, respectively. The polydispersity index (PDI) of all the NPs was below 0.3, indicating excellent dispersibility. Initially, ZIF carried a positive charge with a zeta potential of 10.10 ± 1.15 mV (Fig. 1D), but transfered to a negative charge after Fu encapsulation or HA coating, attributable to electronegative groups of Fu. The elemental composition of HAZ@Fu NPs was shown in Fig. 1B, determined by element mappings of Transmission Electron Microscopy (TEM), confirmed the presence of the specific sulfur element that originated from Fu molecules.

The crystalline structure of NPs was characterized by X-ray diffractometer (XRD). As shown in Fig. 1E, HAZ, ZIF@Fu, and HAZ@Fu exhibited characteristic peaks for a crystalline structure similar to that of ZIF, with 2θ values of 12.8 and 18.25°. The appearance of a novel diffraction peak at 13.8°, coupled with a decreased peak width-to-height ratio in the ZIF@Fu and HAZ@Fu samples, also indicated the successful incorporation of Fu molecules.

Fourier transform infrared spectroscopy (FTIR) analysis of various NPs was conducted. As illustrated in Fig. 1F, both the crude Fu and the Fu (Molecular weight < 10 kDa) showed characteristic peaks at 840, 1049, 1248, and 1630 cm-1, which are attributed to the bending vibrations of C-O-S, stretching vibrations of C-O-C, asymmetric stretching vibrations of S = O bonds in sulfate moieties, and bending vibrations of O-H groups, respectively. The peak intensity at 1220–1270 cm-1 was higher for Fu, indicating a higher content of sulfate esters. ZIF@Fu exhibited corresponding peak changes at approximately 1049, 1248, and 1630 cm-1 compared to ZIF, indicating sufficient Fu loading. Similar changes in FTIR spectra were also observed in the comparison between ZIF and HAZ (Fig. 1G).

The pH-responsive degradation ability of ZIF-8 NPs was verified using the Zinc Colorimetric Assay Kit [49], which determined the concentration of released zinc ions. Considering the in vivo pH measurements of mouse wounds have revealed that wound pH is generally slightly acidic (pH 5.1) at baseline, and tends to shift towards the alkaline range as clinical infection progresses [50]. PBS with pH of 6.0 and 7.4 were specifically chosen to evaluate the sustained release of HAZ@Fu. At 8 h, approximately 82.66% of the Zn^2+^ was released at pH 6.0, while only 56.49% was released at pH 7.4 (Fig. 1H). It suggests that HAZ@Fu NPs retain the pH sensitivity of ZIF-8 and can efficiently release most cargoes in an acidic environment. Furthermore, the CCK-8 assay was used to assess cell viability of RAW 264.7 cells after 24 h treatment with various concentrations of Fu or Fu-loaded NPs (Fig. S1). All MOF-based NPs at a concentration of 80 µg/mL demonstrated satisfactory biocompatibility.

To further determine the antibacterial activity of Fu, ZIF-8, and the Fu-loaded ZIF-8 in vitro, we employed a colony-forming unit (CFU) assay [51] to detect minimum bactericidal concentrations (MBCs). MRSA (> 1 × 10^6^ CFU/mL) were co-cultured with crude Fu, Fu, ZIF, and ZIF@Fu at different concentrations for 16 h. Subsequently, the mixtures were diluted 100-fold with PBS and inoculated onto Luria-Bertani (LB) agar plates, then incubated at 37 °C for 24 h. ZIF@Fu against MRSA was also exmined (Fig. S2). Crude Fu exhibited an unreliable antibacterial effect at a concentration of 7.5 mg/mL. The MBCs of Fu, ZIF, HAZ, ZIF@Fu, and HAZ@Fu were around 30, 75, 75, 30, and 30 µg/mL, respectively. In addition, we detected the zones of inhibition (ZOI) of Crude Fu, Fu, ZIF, and ZIF@Fu against MRSA. As shown in Fig. 1J, crude Fu showed no significant ZOI at high concentrations, whereas the ZOI diameters of Fu, ZIF, and ZIF@Fu (at an equivalent concentration of 3 mg/mL, which contained 90 µg in filter papers) for MRSA were 18.10 ± 0.72, 10.60 ± 0.49, and 20.64 ± 1.08 mm, respectively. The ZOI diameter of the ZIF@Fu group was notably larger than that of the Fu and ZIF group (*p* < 0.05), aligning with the MBCs test results. However, scattered small bacterial colonies still remained at the edges of the ZOI of the Fu group, while no such colonies were present in the ZIF@Fu group. It demonstrated that both Fu and ZIF possess considerable antibacterial effects, while combination of them could decreased the effective antibacterial concentration, potentially due to their combined ability to damage the bacterial capsule, thereby significantly affecting bacterial permeability.

**Table 1:** Hydrodynamic size and zeta potential of all NPs.

Sample	Size (nm)	PDI	Zeta potential (mV)
ZIF-8	116.0 ± 0.7	0.088 ± 0.023	+ 10.10 ± 1.15
HAZ	168.5 ± 1.5	0.151 ± 0.012	-12.10 ± 1.10
ZIF@Fu	222.5 ± 3.6	0.054 ± 0.031	-26.57 ± 0.55
HAZ@Fu	271.1 ± 8.4	0.225 ± 0.013	-38.97 ± 0.47

### Synthesis and characterization of HAZ@Fu MNs

**Fig. 2:**
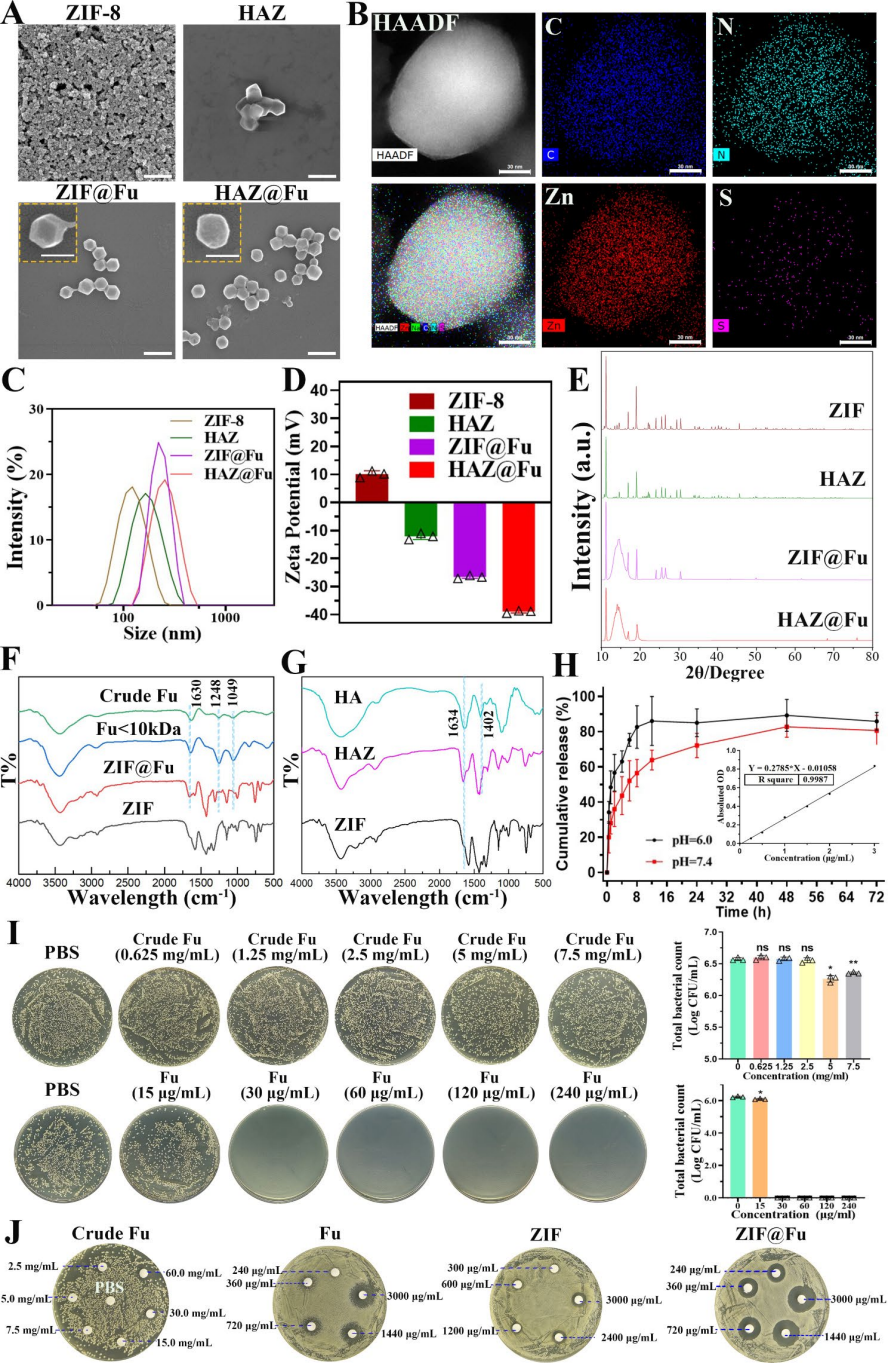
Characterization and antibacterial activity of free Fu, ZIF NPs and Fu-loaded NPs. (**A**) SEM images (scale bar: 500 nm, 200 nm in inset images). (**B**) TEM elemental mappings of HAZ@Fu. (**C**) The size distribution, (**D**) zeta potential, and (**E**) XRD patterns of Zn-MOF derived nanoparticles. (**F**) FTIR spectra of Fu, crude Fu, ZIF and ZIF@Fu. (**G**) FTIR spectra of HA, ZIF and HAZ. (**H**) The release curve of Zn^2+^ derived from the decomposition of ZIF NPs in solution at pH = 7.4 or 5.0 (inset: standard curve of zinc ions). (**I**) MRSA cells (1 × 10^6^ CFU/mL) resuspended in PBS were incubated with crude Fu, Fu, ZIF and ZIF@Fu in vitro for 16 h, serially diluted 100-fold with PBS, inoculated onto LB agar plates, then incubated for another 24 h. (**J**) ZOI test of Crude Fu, Fu, ZIF and ZIF@Fu against MRSA

**Fig. 3:**
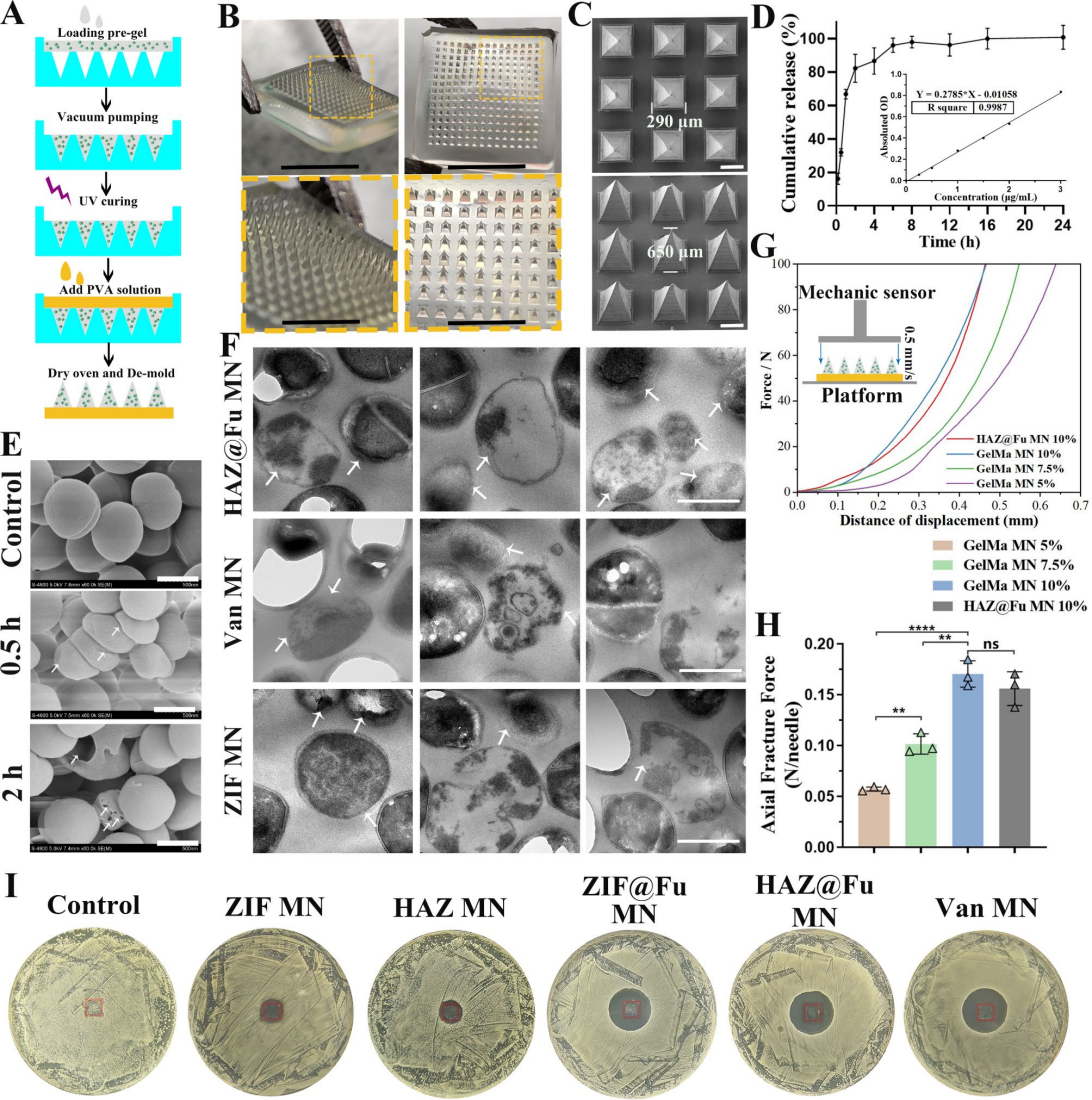
Fabrication and characterization of the HAZ@Fu MNs. (**A**) Schematic illustration of the synthesis of a HAZ@Fu MN patch. (**B**) Isometric and tip-upward photographic images of a HAZ@Fu MN patch, along with the corresponding zoomed-in images (scale bar: 5 mm and 2 mm, respectively). (**C**) SEM images of the HAZ@Fu MN patch. (**D**) Cumulative release of Zn^2+^ from the HAZ@Fu MN patch. (**E**) SEM images of MRSA co-cultured with HAZ@Fu MN in PBS, arrows indicate distorted and disintegrated MRSA (scale bar: 500 nm). (**F**) TEM images of MRSA co-cultured with HAZ@Fu MN, Van MN and ZIF MN in PBS for 24 h, arrows indicate distorted and disintegrated MRSA (scale bar: 500 nm). (**G**) Force‒displacement curves of MNs with different compositions (inset: schematic diagram of the mechanical strength test). (**H**) The axial fracture forces of the different MNs. (**I**) ZOI test of different MNs against MRSA, red rectangle indicated the position of MNs

The biocompatible photocrosslinked GelMA hydrogel was chosen as the material for MN tips, which were loaded with NPs or Van. Van MNs were used as a positive control for evaluated antibacterial efficacy in vitro or in vivo experiment. PVA was selected as the backing layer of MNs due to its biosafety, mechanical properties, and rapid dissolution. The epidermal layer of human skin is typically less than 250 μm in thickness, also considering previous research of MNs, we selected a polydimethylsiloxane (PDMS) MN mold with a needle length of 650 μm (Fig. S3A), and fabricated the MNs by a two-step template replication method [40, 41] (Figs. 2 and 3B).

The Fig. 3B presents a photographic image of the MN patch, which consists of a 15 × 15 array of microneedles. The SEM images verify that the MN tips are pyramidal in shape and are neatly arrayed on the backing layer, as shown in Fig. 3C, the dimensions of the MN tips include a base length of 290 μm and a height of 650 μm. This sharp pyramidal structure allowed for quick, noninvasive skin insertion, as further tested in mouse skin. As shown in Fig. S3B-D, the MN was inserted into the mouse skin. The traces of puncture channels, stained with methylene blue, were visibly present on the skin tissue. Histological analysis using frozen sections stained with H&E also provided in Figure S4, the marked arrows indicate channels produced by the MN insertion breached the stratum corneum barrier and extended into the dermal layer. The chanal spacing corresponds precisely to the shape of the microneedle tips, with penetration exceeding 300 μm into the subcutaneous layer. This confirmed that the MNs possess adequate mechanical strength to penetrate the dermis of the skin effectively. The HAZ@Fu MNs was then co-cultured with MRSA in 1 mL of PBS. SEM was employed to observe the morphological changes in MRSA at various time points. Significant distortions in MRSA were observed within 0.5 h, and bacterial capsules started disintegrate by 2 h (Fig. 3E). TEM was also employed to observe the MRSA after incubated with HAZ@Fu MN, Van MN and ZIF MN for 24 h. As shown in Fig. 3F, it clearly reveals that in all three groups, there is evident destruction of the MRSA capsule structure and lysis of the bacterial cells (indicated by arrows). Both SEM and TEM verified the excellent in vitro antibacterial activity of HAZ@Fu MN.

To examine the release kinetics of the HAZ@Fu MN, the MNs were immersed in 1 mL PBS, the concentration of zinc ions in the released system was measured over time. The release profile demonstrated a sustained release pattern, with a cumulative release rate of 82.27 ± 8.36% detected at 2 h (Fig. 3D). LB agar plates were employed to simulate the localized temperature skin wound tissue by placing them in a 37 °C incubator. Following the application of the MN into the LB agar plates, we observed the degradation of the MNs over various time intervals. When the MN patch was inserted into an LB agar plate at 37 °C, it almost completely dissolved within 2 h (Fig. S5), aligning perfectly with the release profile of the HAZ@Fu MN. Additionally, only 40-50% of zinc ions were released from the ZIF@Fu NPs at the 2h (Fig. 1H), indicating that the NPs possess a more sustained release effect compared to MNs. The combination of MNs with NPs is likely to create a longer-acting, more reliable controlled release system, while the MN structure further ensures an extended duration of action for the NPs.

We evaluated the mechanical properties of the MNs using an electronic tension testing machine to test the maximum strain capacity of their needle tips. The MNs were first placed on a horizontally positioned fixed station with tips facing a force sensor that slowly approached the MNs. The force measurements began when the sensor touched the MN tips and continued until the measured value reached 100 N. The force‒displacement curve of each sample was recorded (Fig. 3G), with the Young’s modulus and the Axial Fracture Force obtained when the sensor traveled 0.3 mm, serving as the stiffness index of the MNs (Fig. S7A and Fig. 3H). The Young’s modulus of MNs was increased with the increase of GelMA concentration, indicating the enhancement of the mechanical strength of the MNs (*p* < 0.05). An increase in the GelMA concentration led to an increase in the Young’s modulus of the MNs, indicating an enhancement in their mechanical strength (*p* < 0.05). At a GelMA concentration of 10%, the MNs could withstand compressive forces of 0.17 ± 0.013 N/needle, strong enough to successfully puncture the skin [52]. The loading of NPs did not significantly alter their mechanical strength (*p* > 0.05). To evaluate the antibacterial activity of the drug-loaded MNs, the ZIF MNs, HAZ MNs, ZIF@Fu MNs, HAZ@Fu MNs, and Van MNs were incubated with MRSA at 37 °C for 24 h. As observed in Fig. 3I and Fig. S7B, ZOI in MRSA were caused by ZIF@Fu MN, HAZ@Fu MN, and Van MN, with diameters of 22.27 ± 0.40, 23.56 ± 0.67, and 25.02 ± 0.59 mm, respectively. In contrast, both ZIF MN and HAZ MNs were only able to inhibit bacterial growth within the patch coverage area, and their ZOI diameters were significantly smaller than those of the ZIF@Fu MN, HAZ@Fu MN, and Van MN groups (*p* < 0.05). Bare MNs did not exhibit any antibacterial effect. Consequently, the antibacterial potency of the HAZ@Fu MNs was robust, approaching that of the Van MNs.

### Cellular uptake efficiency of NPs

**Fig. 4:**
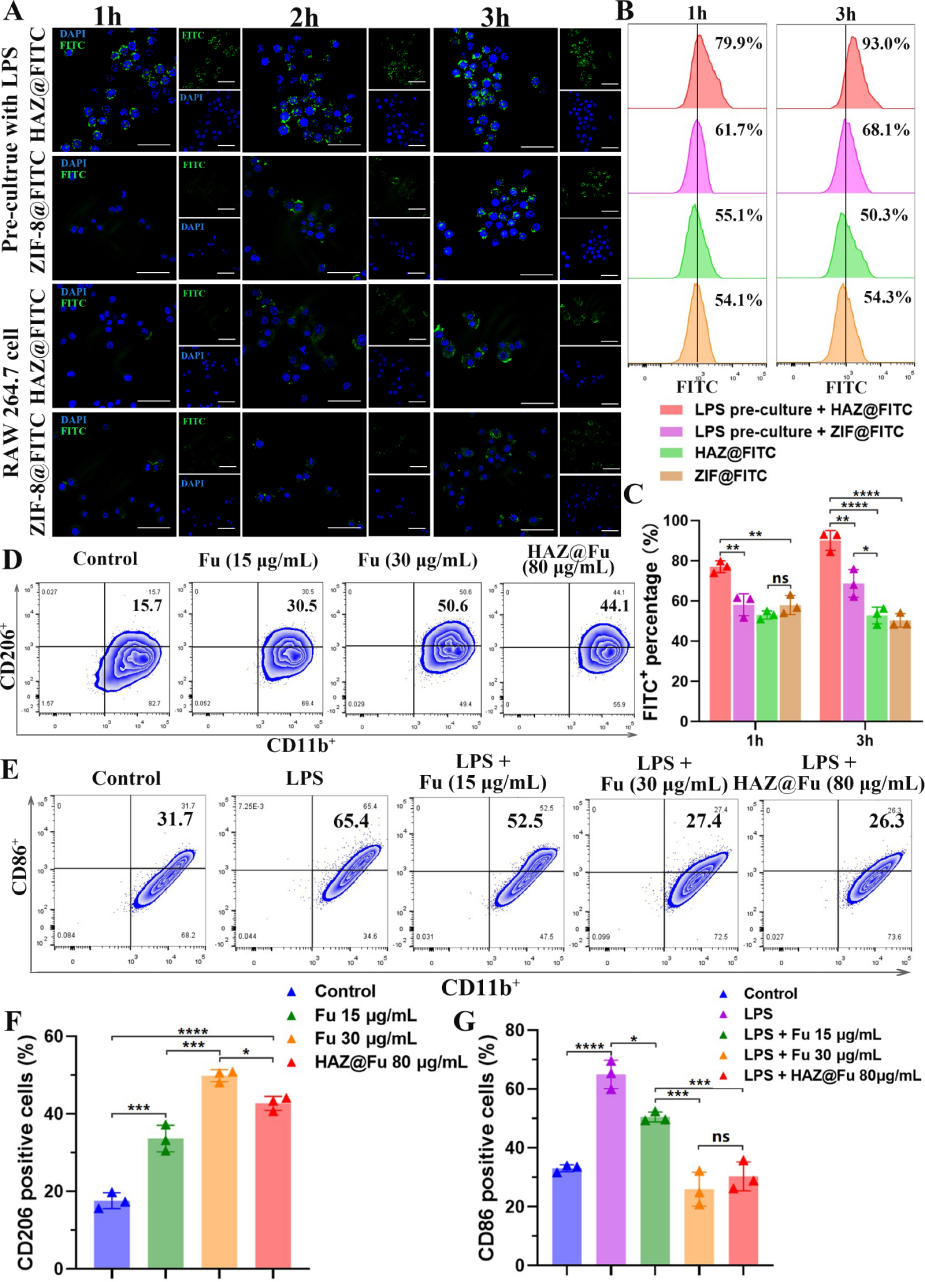
(**A**) Confocal microscopy images of FITC-loaded NPs uptake by untreated RAW cells or RAW cells precultured with LPS for 1, 2 and 3 h (scale bar: 50 μm). (**B**) The FCM results of the FITC signal to demonstrate the uptake efficiency of NPs at 1 and 3 h, stained with FITC and F4/80. (**C**) The FITC-positive percentage of cells cultured with FITC-loaded NPs after 1 and 3 h. (**D**) The FCM results of BMMs cultured with normal medium and medium cotaining Fu (15 and 30 µg/mL, repectively) or HAZ@Fu NPs (80 µg/mL) for 24 h, stained with the M2 marker CD206, and macrophage markers CD11b (gated by F4/80). (**E**) FCM results of BMMs cultured with normal medium and medium containing LPS (100 ng/mL), LPS + Fu or LPS + HAZ@Fu NPs stained with the M1 marker CD86 and macrophage marker CD11b (gated by F4/80). (**F**) Ratio of BMMs stained with M2 marker CD206. (**G**) Ratio of M1 phenotype BMMs stained with M1 marker CD86

To evaluate the uptake efficiency of HAZ@FITC NP in both the M1-polarized RAW cells and the M0 RAW cells, lipopolysaccharide (LPS) was used to activate macrophages into the M1 phenotype. The rates of M1 polarization (indicated by CD86^+^) were tested by flow cytometry (FCM). As illustrated in Fig. S8, the levels of the main M1 marker, CD86, significantly elevated after treating RAW 264.7 cells with LPS (100 ng/mL) for 24 h, increasing from 22.6 to 65.8%. As shown in Fig. 4A, only the M1 polarized RAW cells + HAZ@FITC group displayed noticeable FITC signals at 1 h. The FITC fluorescence signal was predominantly localized around the DAPI-stained nuclei, indicating rapid internalization of the nanoparticles into the cytoplasm of the cells. Among all the groups, the M1 polarized RAW cells in the HAZ@FITC group exhibited the highest FITC fluorescence signal intensity. Moreover, the fluorescence intensity of all groups increased from 1 to 3 h, suggesting the effect was time-dependent.

The uptake efficiency was further quantified using FCM (Fig. 4B, C). The M1 polarized RAW cells + HAZ@FITC group also demonstrated the highest FITC fluorescence intensity (77.10 ± 2.91% at 1 h and 90.20 ± 4.94% at 3 h). In contrast, the M1 polarized RAW cells + ZIF@FITC group only achieved 58.23 ± 5.49% at 1 h and 68.8 ± 6.88% at 3 h. These results suggest that an upregulation of CD44 expression in M1 polarized cells allows for the increased uptake of HA-coated nanoparticles.

### Effects of Fu and HAZ@Fu NPs on the polarization of macrophages

The polarization of Bone marrow-derived macrophages (BMMs) was also investigated by detecting the M1 marker CD86 and the M2 marker CD206 using FCM. Figure 4D and F reveal that after 24 h of co-culture, the M2 phenotype of BMMs was activated by Fu and Fu-containing NPs. The percentages of CD206 positive cells after cultured with Fu at concentrations of 15 µg/mL (33.67 ± 3.42%), 30 µg/mL (49.87 ± 1.54%) and with 80 µg/mL HAZ@Fu NPs (42.73 ± 1.80%), which were notably higher than that of control group (17.63 ± 2.06%, *p* < 0.05). Furthermore, Fu or HAZ@Fu NPs could inhibit the M1 polarization of BMMs induced by LPS. As displayed in Fig. 4E and G, the percentage of CD86 positive cells after cultured with LPS (100 ng/mL for 24 h) was 65.00 ± 4.81%, while in the LPS + 15 µg/mL Fu group was 50.53 ± 1.71%, in the LPS + 30 µg/mL HAZ@Fu NPs group was 25.97 ± 5.77% and in the LPS + 80 µg/mL HAZ@Fu NPs group was 30.33 ± 4.91%. The FCM test was also conducted after BMMs co-cultured with HAZ@Fu NPs (40 and 80 µg/mL) for 48 h, as shown in Fig. S9, The presence of CD206 positive cells was 16.6% in the control group. After coculture with the HAZ@Fu for 48 h, there was also a marked increase in the population of CD206 positive cells. In the group treated with 40 µg/mL of HAZ@Fu, the percentage of CD206 positive cells rose to 26.2%, and in the group treated with 80 µg/mL of HAZ@Fu, it increased significantly to 51.5%.

Both Fu and Fu-loaded NPs facilitate M2 polarization and inhibit M1 polarization of macrophages in a dose- and time-dependent manner. Remarkably, 80 µg/mL HAZ@Fu NPs with approximate 10.48 µg/mL Fu match the M1 inhibitory effect of 30 µg/mL free Fu, likely due to enhanced HA-coated NP uptake by M1 macrophages or intrinsic activity of HA.

### Gene expression profle of BMMs analyzed by microarray

**Fig. 5:**
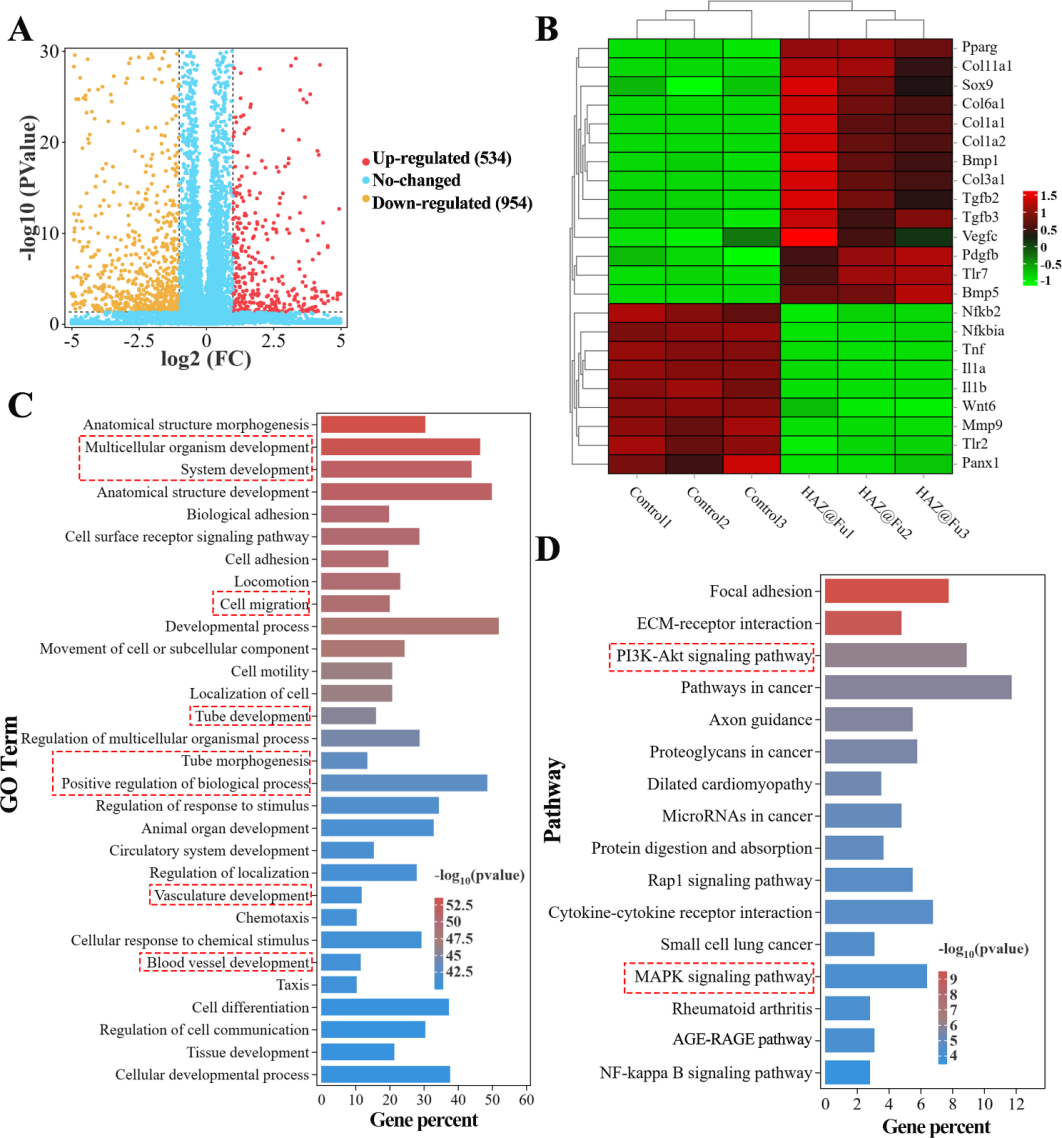
RNA sequencing was employed to detect the whole mRNA expression of BMMs cultured with HAZ@Fu NPs or normal culture medium for 24 h. (**A**) Volcano plot showing the Gene expression differences between the HAZ@Fu and Control group. (**B**) Microarray heat map depicting the fold change of selected genes expression. (**C**) The 30 main GO terms in the HAZ@Fu group compared with those in the control group, with the highest enrichment levels were selected based on the p-value. (**D**) Representative 16 related pathways analyzed by KEGG pathway enrichment method

RNA sequencing was employed to detect the whole mRNA expression of BMMs cultured with or without HAZ@Fu NPs for 24 h. As shown in Figs. 5A and 1488 differentially expressed genes (DEGs) between the HAZ@Fu and control group were identified, with 534 genes upregulated and 954 genes downregulated in HAZ@Fu cluster (more than 2-fold changes in expression). From these genes, we identified those related to inflammation (such as IL-1α, IL-1β, TNF, MMP, and NFκB) and tissue repair (including Pparg, Col1a, VEGF-c, and TGF-β) as relevant to our study. We then created heat maps to visually represent the expression levels of these selected factors (Fig. 5B). The outcomes revealed that in the HAZ@Fu group, anti-inflammatory factors, anti-inflammatory pathway proteins, and tissue repair-related factors were highly expressed, while inflammatory factors or inflammatory pathway proteins were expressed at lower levels. As demonstrated in Fig. S10A, the Gene Ontology (GO) function enrichment analysis indicated that DEGs were primarily enriched in biological processes (BP). Subsequently, a specific GO function enrichment of BP was conducted, with the top 30 most enriched GO terms in the HAZ@Fu group compared with the control group, indicating that DEGs were enriched developmental process-related BP, including cell migration, tube morphogenesis and tube/vasculature/blood vessel development (Fig. 5C). The reactome enrichment analysis revealed DEGs predominantly enriched in extracelluar matrix organization and collagen formation (Fig. S10B). CD 206 positive cells.

To further understand the signaling path ways involved in regulating macrophage phenotype switching and tissue regeneration gene expression, kyoto encyclopedia of genes and genomes (KEGG) pathway analysis was applied. Figure 5D revealed the main 16 related pathways enriched between HAZ@Fu and control group. Specifically, the NF-κB signaling pathway, which is associated with the M1 phenotype, showed a slight downregulation, the PI3K-Akt signaling pathway, a key pathway regulating M2 polarization, anti-inflammatory responses, and angiogenesis [53, 54], was signifcantly enriched by KEGG analysis. In brief, the RNA sequencing data suggested that HAZ@Fu can instigate a transformation in the gene expression profile of macrophages towards the M2 phenotype, promoting collagen deposition, tissue regeneration, and angiogenesis. The KEGG analysis indicates these functions are likely accomplished through the PI3K-Akt pathway, although further validation is certainly required.

### Intracellular fate and intracellular activity of ZIF and HAZ NPs

To explore the potential colocalization of HAZ NP with lysosomes, given that the uptake of NPs in RAW 264.7 cells was observed to rise progressively over the initial 3 h, RAW 264.7 cells were incubated with either ZIF@FITC or HAZ@FITC NPs for 4 h, after that, the cells stained with Lyso-tracker Red DND-99. Live cell images were then recorded using confocal laser scanning microscopy (CLSM). In addition, the Plot Profile feature of ImageJ software was utilized to describe the distribution of the fluorescence signal. The Pearson’s correlation coefficient (PCC) is the metric generally used to quantify the degree of colocalization between different fluorescence signals. The PCC between different fluorescence signals was determined based on a total of 6 images derived from three independent experiments.

**Fig. 6:**
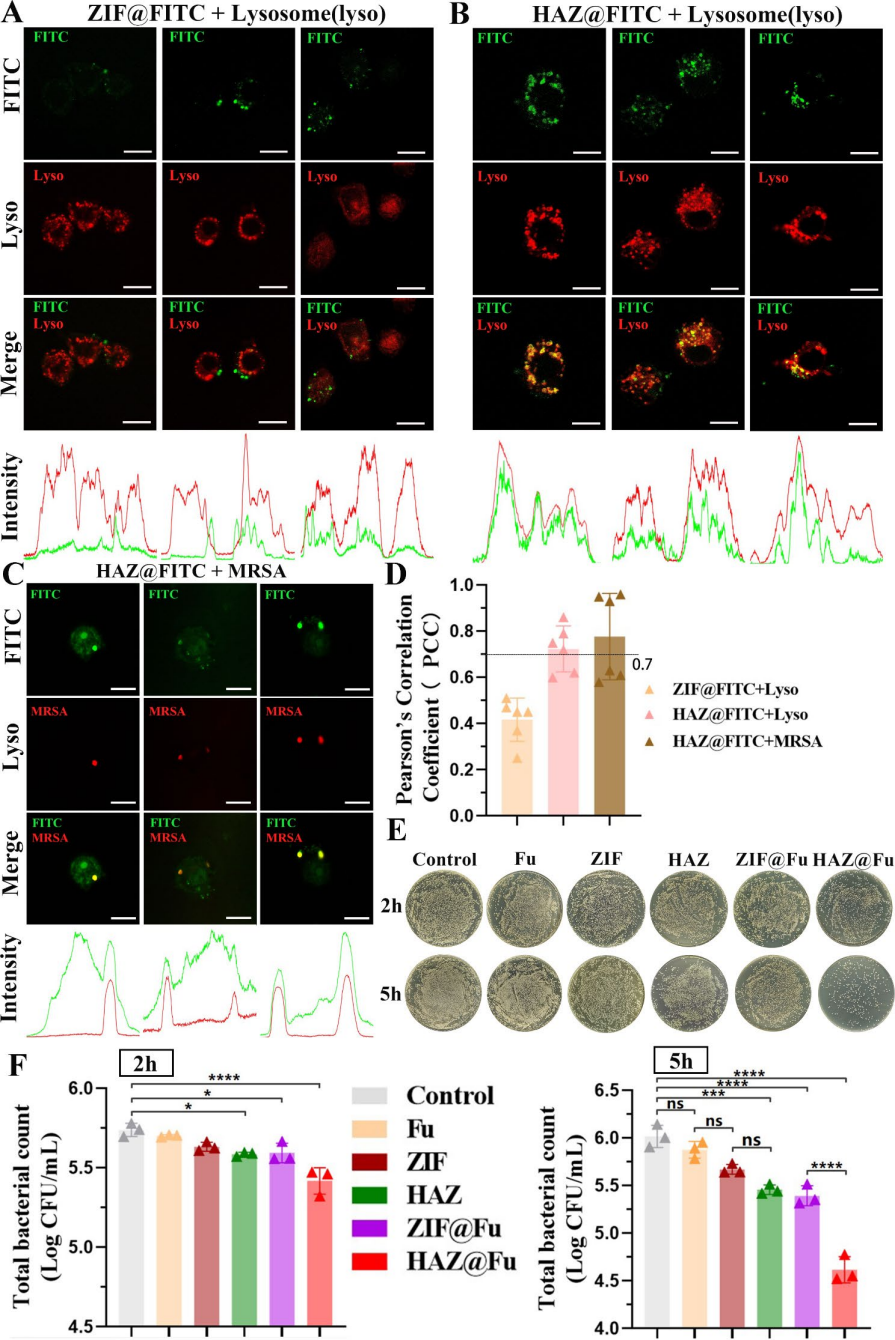
(**A**) Colocalization of ZIF@FITC NPs with lysosomes. RAW 264.7 cells were incubated with 60 µg/mL FITC-loaded NPs for 4 h. Then the slides were rinsed with PBS and treated with LysoTracker Red for 5 min at 37 °C. Images were immediately captured from live cell (scale bar: 10 μm). (**B**) Colocalization of ZIF@FITC NPs with lysosomes (scale bar: 10 μm). (**C**) Colocalization of HAZ NPs with intracellular MRSA. RAW 264.7 cells were incubated with MRSA (marked by mCherry). After which extracellular bacteria were removed, the cells were then rinsed with PBS and further incubated with 80 µg/mL HAZ@FITC NPs for 3 h. Images were captured using CLSM (scale bar: 10 μm). (**D**) PCC was calculated using ImageJ. (**E**) Infected RAW 264.7 cells were incubated with Fu, ZIF, HAZ, ZIF@Fu, HAZ@Fu, and PBS for 2 and 5 h. Subsequently, all cells were lysed, and 100 µL of each lysate (10-fold dilution) was cultured on LB agar plates and incubated for 24 h. Digital images of the plates were captured. (**F**) Viable cell counts were calculated using three biological replicate count data (each derived from three technical replicate data). All data shown are viable counts multiplied by 100 (dilution multiple), with log10 transformed

As illustrated in Fig. 6A and B, a significant correlation was observed between the HAZ@FITC signal and lysosomes, the intensity images showed a high concurrence between the peaks of the red and green fluorescence signals. Conversely, the ZIF@FITC group did not display a significant correlation with lysosomal fluorescence, yielding a PCC value of 0.42 ± 0.09. These findings affirm that HAZ NPs are capable of accumulating in the lysosomes of RAW 264.7 cells.

To further explore the capability of HAZ NPs to target intracellular MRSA at a subcellular level, the MRSA (USA 300) were labeled with green fluorescent protein (GFP) or mCherry for the fluorescent tracking. RAW 264.7 cells infection model was established by coculturing with MRSA for 1 h, and using FBS-free DMEM containing Van at a concentretion of 2×MIC to remove any extracellular bacteria. The infected cells were cocultured with HAZ@FITC for 3 h and washed twice with PBS, then observed by CLSM immediately (Fig. 6C and Fig. S11A). It also find a significant correlation between the FITC signal and MRSA, indicated by the PCC values above 0.7 for both lysosomes (0.72 ± 0.10) and MRSA (0.77 ± 0.19) when colocalized with FITC-labeled HAZ nanoparticles. Additionally, the colocalization of MRSA (labelled with GFP) with lysosomes was also detected by CLSM (Fig. S11B), it demonstrated that nearly all the internalized bacteria were found in the lysosomes within the cells (indicated with arrows). This accumulated evidence strongly suggests the high efficiency of HAZ NPs in targeting intracellular MRSA in macrophages.

To evaluate the intracellular activity of HAZ@Fu NPs, RAW 264.7 cells infection model were treated with Fu, ZIF NPs, HAZ NPs, ZIF@Fu NPs, and HAZ@Fu NPs (all groups at an equivalent concentration of 60 µg/mL) for 2 or 5 h. Subsequently, cells were lysed using 0.025% Triton X, and lysates were collected and serially diluted with sterile PBS, then inoculated onto LB agar plates, and incubated for 24 h. As shown in Fig. 6E and F, after 2 h of incubation, HAZ@Fu exhibited the most significant antimicrobial activity (*p* < 0.05). After 5 h of incubation, both ZIF@Fu and HAZ@Fu displayed significant antimicrobial activity against intracellular MRSA. While the pure Fu showed a limited effect on intracellular MRSA, both ZIF and HAZ showed higher efficacy compared to the control group (*p* < 0.05). The high efficiency of HAZ@Fu in treating intracellular MRSA could be attributed to: I) The optimal size of NPs facilitating cell uptake, II) The antibacterial effects of both Fu and ZIF, with their combination in the drug-loading system producing a synergistic effect and thereby reducing the effective antibacterial concentration, and III) The HA coating providing specific intracellular bacterial targeting.

### Evaluation of healing in MRSA-infected wounds in vivo

**Fig. 7:**
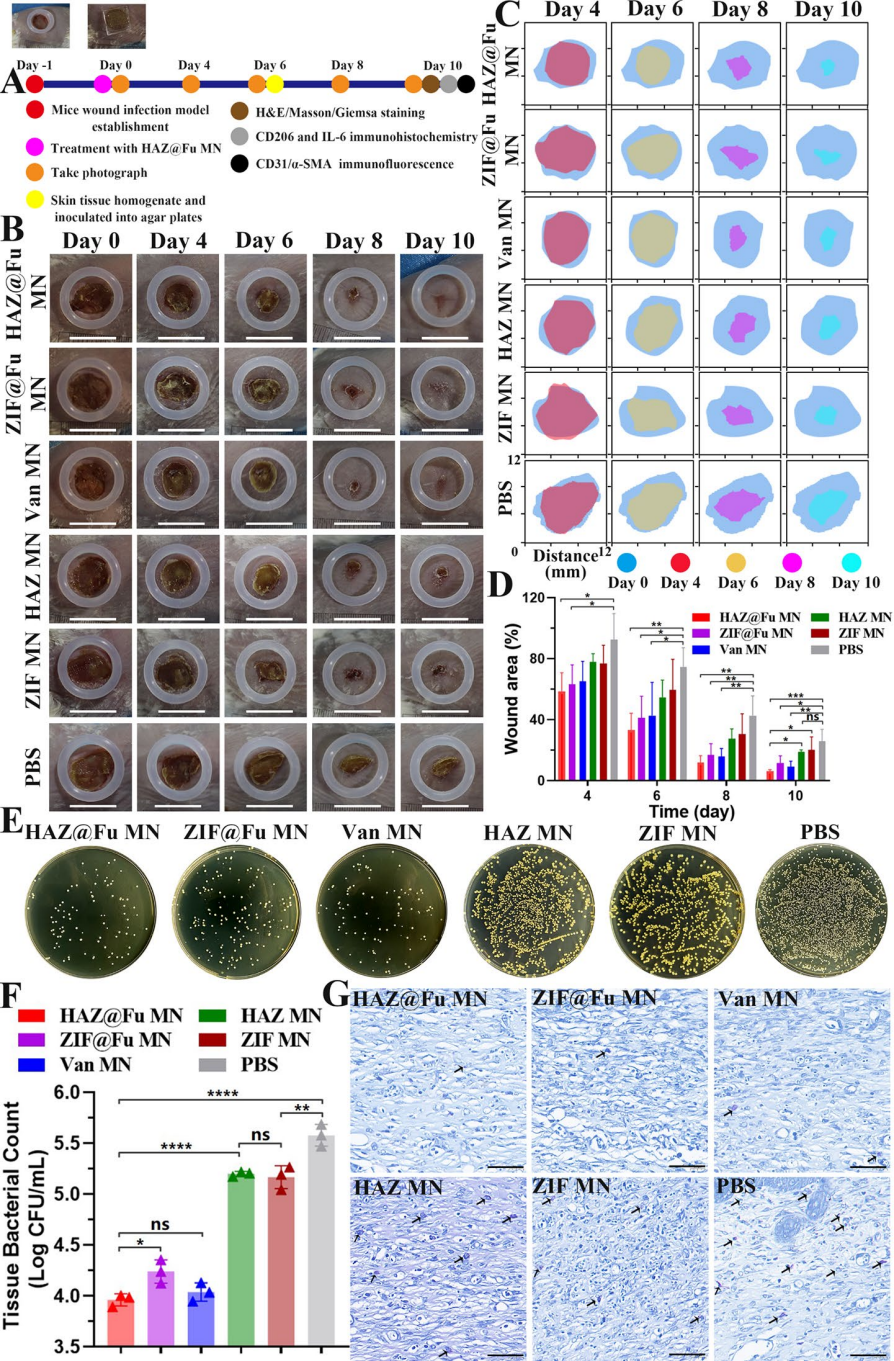
Treatment efficiency of the MNs on MRSA-infected wounds. (**A**) Illustration of the experimental procedures for in vivo treatment of infected wounds. (**B**) Representative photographs of wounds with various treatments at different time points. As a reference, a rubber ring with an inner diameter of 1 cm was positioned around the wound (scale bar: 10 mm). (**C**) Simulation of wound morphological transformations over a span of 10 days. (**D**) Comparative analysis of the relative wound area among different groups over 10 days. (**E**) Representative photographs and (**F**) Corresponding histograms of bacterial colonies derived from MRSA-infected tissue homogenates after undergoing diverse treatments on Day 6. G) Giemsa staining of wound tissues post-10 days treatment (scale bar: 50 μm). Bacteria are indicated by arrows

In vivo experiments were conducted in female BALB/c mice between 6 and 8 weeks. To establish a full-thickness infected cutaneous defect mouse model, a round wound with 0.8 cm were created on the back of mice, and 50 µL PBS containing MRSA were subsequently inoculated. The mice were randomly divided into 6 groups. The group without any treatment served as the control. The other five groups were treated with ZIF MN, HAZ MN, ZIF@Fu MN, HAZ@Fu MNs, and Van MN (pure Van loaded into MNs), respectively. MNs were applied directly to the wounds with appropriate pressure to enable the microneedles to penetrate the skin, and the dissolution process was also observed. As shown in Fig. S6, 15 min after the treatment, the contact surface became moist, indicating that the GelMA hydrogel needles had started to dissolve noticeably. Subsequently, at 60 min, the backing layer became visibly damp and began to degrade. At 150 min only the residual shapes of the microneedle bases were left, and by 300 min, a gel-like substance remained on the surface of the wound, creating a protective layer. Compared to the dissolution on LB plates, the PVA in the backing layer dissolved more slowly, This is likely attributed to the lower temperature of the backing layer, resulting from the lack of direct contact with the skin, as opposed to the stable temperature and humidity conditions present on the LB plates. Nevertheless, the area of skin contact with the microneedles also became rapidly moist, indicating that the microneedles dissolved quickly. The entire experimental procedure is illustrated in Fig. 7A.

Dynamic changes in wound morphology in Fig. 7B and C revealed that the wound in both the HAZ@Fu MNs and Van MNs groups were nearly complete healing after 10 days. While the wounds in the ZIF MNs, HAZ MNs, and control groups exhibited a slower healing process, with visible scabs remaining. Measurements and analysis of the wound closure rate for each group were conducted using digital imaging techniques (Fig. 7C and D). On day 4, the relative wound areas in the HAZ@Fu MNs and ZIF@Fu MNs groups were 58.60 ± 12.18% and 63.11 ± 12.91%, respectively, and further reduced to 12.08 ± 4.38% and 16.9 ± 7.32% on day 8, representing a significantly greater healing rate than that of the control group (42.74 ± 12.95%). The epidermal scabs of the HAZ@Fu MNs, ZIF@Fu MNs, and Van MNs groups were completely shed, indicated a completed epithelial tissue regeneration process. HAZ@Fu MNs groups achieved a wound closure rate exceeding 90%, significantly higher than both the HAZ MNs and the control group (*p* < 0.05). It demonstrated that HAZ@Fu possesses effective antibacterial activity and also promotes tissue regeneration in wounds.

The in vivo antibacterial efficiency was also evaluated. On day 6, skin tissue containing the entire wound area were collected from all groups for tissue homogenization. The homogenate was subsequently serially diluted with PBS and inoculated onto LB agar plate, incubated for 24 h and the viable cell counts were determined. As illustrated in Fig. 7E, a noticeable decrease in the number of colony-forming units was observed after treatment with HAZ@Fu MNs and Van MNs, and no substantial difference was noted between them (*p* > 0.05). However, ZIF@Fu MNs group displayed a lower bacterial killing efficiency than Van MNs group in vivo (*p* < 0.05). As shown in Fig. 7G, after ten days of treating the wounds, Giemsa staining was performed to visualize bacteria in the tissue sections. Consistent with the bacterial counts from the tissue homogenates, abundant bacteria were observed in the tissues of the control, HAZ MNs, and ZIF MNs groups.

**Fig. 8:**
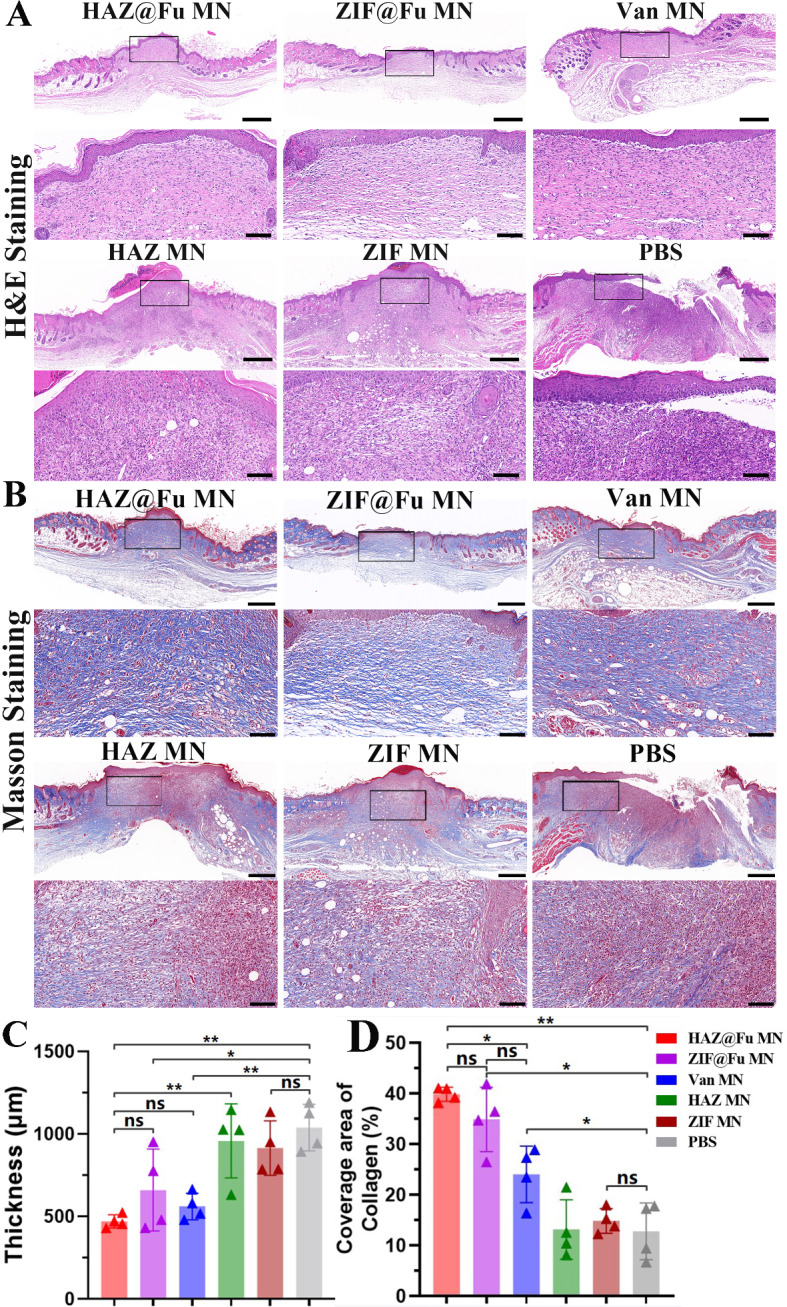
Evaluation of MRSA-infected wound healing in Vivo. (**A**) Optical images and the corresponding magnified images of H&E staining (scale bar: 100 μm, 500 μm after magnification). (**B**) Optical images and the corresponding magnified images of Masson staining (scale bar: 100 μm, 500 μm after magnification). (**C**) Quantitative analysis of granulation tissue thickness observed in the H&E staining across six groups. (**D**) Quantitative analysis of the coverage area of collagen as observed in Masson staining

To examine the reconstruction of epidermal tissue, histological tests were conducted on Day 10. Hematoxylin and eosin (H&E) staining was employed to investigate the regeneration of wound beds, the formation of granulation tissue, and the processes of epithelial formation. The thickness of the granulation tissue in each group were also measured (Fig. S10). The Fig. 8A revealed that a new stratum corneum was fully formed in the HAZ@Fu MNs, ZIF@Fu MNs and Van MNs groups. The thickness of the granulation tissue was significantly reduced compared to that of ZIF MNs, HAZ MNs and control group. Moreover, a considerable number of inflammatory cells, which identified by their high nucleus-to-cytoplasm ratio and dominant nuclear staining, were noted within the wound area and remaining scabs in ZIF MNs, HAZ MNs and control group.

Quantitative analysis revealed a significant reduction in the thickness of granulation tissue with HAZ@Fu MNs treatment (440.60 ± 20.36 μm) in comparison to the control (997.80 ± 170.30 μm) and the ZIF MNs or HAZ MNs groups (Fig. 8C). It suggested that HAZ@Fu MN group present improved regulation of inflammation and accelerated tissue regeneration. Processes like collagen synthesis, deposition, and directional alignment, which are crucial for tissue remodeling, also play a significant role in wound healing, and masson staining was carried out to observe the collagen formation and deposition. Figure 8B showed a significant area of newly formed collagen, characterized by its directional alignment and a deep blue stain, evident in the neotissue of the epithelium in both the ZIF@Fu and HAZ@Fu groups. Correspondingly, the extent of collagen coverage was quantified as 39.87 ± 1.41% in the ZIF@Fu group and 35.18 ± 5.92% in the HAZ@Fu group, respectively. Which significantly higher than that in the Van MNs group (24.08 ± 5.59%, *p* < 0.05). These findings suggest an increased amount of collagen deposition and improved tissue remodeling.

## Evaluation of tissue inflammation and angiogenesis following treatment

To further examine the anti-inflammatory and angiogenic phenotypes within the tissue. The CD206 and IL-6 immunohistochemistry staining, as well as double immunofluorescence staining of CD31 and α-smooth muscle actin (α-SMA) were conducted to skin tissue slices.

**Fig. 9:**
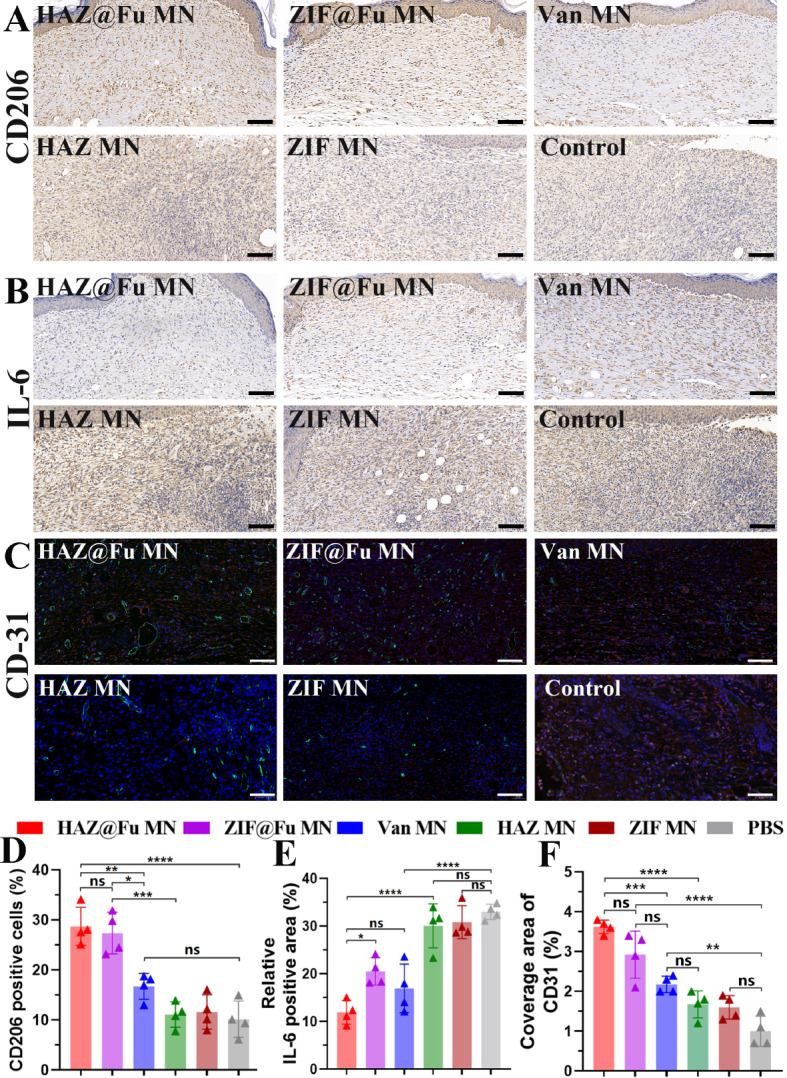
Evaluation of tissue regeneration and anti-inflammatory effects following MNs treatment. (**A**) Immunohistochemistry staining of M2 macrophage maker CD206 and (**B**) inflammatory marker IL-6, respectively (scale bar: 100 μm). (**C**) Dual immunofluorescence staining of CD31 (green) and α-SMA (red) for neovascularization analysis (scale bar: 100 μm). Quantitative evaluation of (**D**) CD206-positive cells, E) the relative IL-6-positive area and F) the relative coverage area of CD31, repectively

As shown in Fig. 9A and B, the control group and the single MOF MNs groups demonstrated high levels of secreted IL-6 and a small population of CD206 positive cells, suggesting a persistent inflammatory response in these groups. In contrast, the HAZ@Fu MNs group showed minimal IL-6 secretion and numerous CD206-positive cells, indicative of reduced inflammation (Fig. 9D, E). The ZIF@Fu MN group, another Fu-loaded MOF MNs group, exhibited more IL-6 secretion and fewer CD206-positive cells, underscoring the positive effect of HA in treating MRSA-infected wounds.

The degree of neovascularization, another critical healing indicator, can be evaluated through CD31 (endothelial cell markers) and α-SMA (fibroblast markers) immunofluorescence staining. Figure 9C and F reveal a significant increase in new blood vessel density within the wound bed in the HAZ@Fu and ZIF@Fu MNs groups.

The HAZ@Fu group and ZIF@Fu MNs group exhibited CD31 coverages of 3.63 ± 0.17% and 2.93 ± 0.59%, respectively. While Van MNs group exhibited minimal IL-6 secretion, moderate population of CD206 positive cells and moderate CD31 coverage, with percentages of 16.96 ± 5.08%, 16.73 ± 2.59%, and 2.18 ± 0.21%, respectively. It indicated the beneficial effect of HAZ@Fu NPs in modulating the inflammatory response and enhancing neovascularization. In contrast, a lower density of CD31 markers was observed in the control group and the single MOF MNs group, likely due to the inability of a single MOF to completely eradicate MRSA and the persistent MRSA infection may have consequently hindered neovascularization at the wound site.

In summary, the HAZ@Fu MNs group not only demonstrated an anti-MRSA effect similar to the Van MNs group, but also showed superior results in terms of wound closure, epithelial regeneration, neovascularization, and anti-inflammatory effects compared to all other groups.

## Conclusions and limitations

In summary, we have developed MNs capable of penetrating the epidermal barrier, efficiently delivering drugs subcutaneously with rapid in situ dissolution. Upon administration, the MNs not only release the NPs but also form a protective gel-like layer. The diverse biological functionalities of HAZ@Fu nanoparticles are fully actualized in vivo utilizing the MNs delivery system in a mouse model, thereby demonstrating the synergistic effect of the HAZ@Fu NPs-integrated MNs for enhanced therapeutic efficacy. This innovative patch incorporates several strategies into one application, including: I) Efficient transdermal drug delivery and sustained release facilitated by MNs, II) Independent anti-MRSA effects of Fu and ZIF-8, with HAZ@Fu achieving a lowered effective antibacterial concentration through synergistic action, III) HAZ@Fu has the ability to target and eradicate intracellular bacteria at a subcellular level, particularly within lysosomes, and IV) The promotion of tissue regeneration, anti-inflammation, and neovascularization is facilitated by HAZ@Fu NPs.

Our study has adequately demonstrated the targeting capability of HAZ nanoparticles against subcellular MRSA and the lysosomes, and the feasibility of HAZ@Fu in combating intracellular infections in vitro cellular experiments. However, these effects could not directly reflect in in vivo experiments, despite the HAZ@Fu group indeed achieved better therapeutic outcomes compared to the ZIF@Fu group. Through RNA sequencing and subsequent gene enrichment analysis, we have identified that the anti-inflammatory and reparative biological functions of HAZ@Fu may be mediated through the PI3K-Akt pathway. However, this preliminary conclusion will require further validation with additional molecular experiments.

To the best of our knowledge, the study presents the first instance for HA-modified MOF has been employed in an infection model, targeting and eliminating bacteria at a subcellular level. Despite numerous reports on the antimicrobial effects of Fu, this is also the first time the antimicrobial effect of low molecular weight fucoidan has been systemically confirmed, and combined with MOF material to apply in infection treatment.

## Methods

### Materials, cell lines and animals

Zinc nitrate hexahydrate (Zn(NO_3_)_2_·6H_2_O), 2-methylimidazole (2-MIM), hydrogen peroxide, Fucoidan (from Undaria pinnatifida, ≥ 95%, Fu), polyvinyl alcohol (PVA, Mw = 30,000–70,000), Polyvinyl pyrrolidone (PVP, Mw = 40,000), and hyaluronic acid (Mw = 10,000) were obtained from Sigma-Aldrich (USA), Gelatin methacryloyl (GelMa) and Photoinitiator Lithium Phenyl ( 2,4,6-trimethylbenzoyl ) phosphinate (LAP) were obtained from Engineering For Life (Suzhou, China). fluorescein isothiocyanate (FITC), Lyso-tracker Red DND-99, Lyso-tracker Green DND-26 and DAPI were obtained from US Everbright (Suzhou, China). Antibodies of F4/80, CD11b, CD86, CD206 for FCM was obtained from Biolegend (USA). CCK-8 Kit and Zinc Colorimetric Assay Kit were obtained from Elabscience Ltd (Wuhan, China). The macrophage colony stimulating factor (M-CSF) and LPS were provided by Thermo Fisher (USA). Antibodies of IL-6, CD206, CD31, and α-SMA for histological staining were purchased from Wuhan Servicebio Technology Co., Ltd (Wuhan, China). MN patch mold was purchased from Henan Micro-Nano BenTeng Biotechnology Co., Ltd (Henan, China) which was composed of a 15 × 15 arrays and produced 290 μm × 290 μm × 650 μm (W × L × H) MN tips. The S.aureus strains, MRSA ATCC 43,300 and USA 300 (transferred by GFP or meCherry) and RAW 264.7 cells were obtained from was provided by Changsha Plant Biotechnology Co., ltd. After growing overnight at 37℃ in LB medium, the bacteria were shaken at 37℃ until reaching the logarithmic growth phase. Bacteria were washed with PBS three times after centrifugation, McFarland density reached 0.5 was corresponds to MRSA concentration of 0.75 × 10^8^ CFU/mL. Finally, 1 mL of bacterial suspension was used for cell infection or further use. BMMs were obtained from femurs and tibias of 8–12 weeks old mice and cultured as previously described [55]. Then cultured for 7–9 days with 1640 RPMI medium (10% FBS), 1% penicillin/streptomycin and 25 ng/ml M-CSF.

Female BALB/c mice (6–8 weeks old) were used to establish an infectious wound model. All experiments involving the use of animals were performed in accordance with the relevant guidelines and regulations approved by the Ethics Committee at Xiangya Hospital of Central South University in China (2,022,111,417).

### Characterization

The bright-field microscopic images of MNs were observed by an Oplenic digital camera. The microstructures of MN patch and MOF derived nanoparticles were photographed by SEM (Hitachi H-7600). The size distribution, PDI and zeta potential of the NPs (dispersed in DI water) was analyzed by a DLS (Zetasizer Nano ZS, Malvern Instrument, UK) at room temperature (20 °C). The element composition of microparticles was conducted by energy-dispersive spectrum (EDS, FEI Titan G2 60–300). The characteristic peaks of Fu, HA and MOF derived NPs were collected by Fourier Transform Infrared Spectroscopy (FTIR, Spectrum Two, Perkin Elmer). The diffraction peak of NPs was tested by XRD (Ultima IV). The uptaken of NPs and Fluorescence colocalization in cells were observed by CLSM (Zeiss LSM 510). The mechanical strength of MNs was measured through using an electronic universal testing machine (Instron 5982). The OD values of the CCK-8 and Zinc Colorimetric Assay Kit assay were read by a microplate reader (SYNERGY| HTX).

### Preparation of low molecular weight fucoidan

Preparation of low molecular weight fucoidan (Mw < 10 kDa) was performed according to previous study [15]. First, 100 mg crude fucoidan was depolymerized with 30 mL of 3% hydrogen peroxide under magnetic stirring at 60℃ for 6 h. Sodium bicarbonate was added until the pH reached 7.0 to terminate the reaction. The depolymerized fucoidan was then separated by 10 kDa MWCO ultrafiltration centrifuge tube, further purifying by dialyzed with 1,000 MWCO dialysis tubing. The obtained LMWF were weight after lyophilization for further use. In this study, all LMWF used in vivo and in vitro with a molecular weight lower than 10 kDa, therefore, before and after depolymerization, they are collectively referred to as Crude Fu and Fu.

### Fabrication of ZIF-based NPs

ZIF@Fu NPs were synthesized using a facile one-pot biomineralized method as previously described [27]. First, 5 mL of 2-MIM aqueous solution (0.1 mol/L) was mixed with 4 mL PVP aqueous solution (w/v = 3%) for 10 min. 1 mL Zn(NO_3_)_2_ aqueous solution (0.1 mol/L) mix with 6 mg Fu and stirring in an ice bath for 10 min, then slowly dropped into 2-MIM mixture under vigorously stirred, further react under ultrasonic for half an hour. The NPs were separated by centrifugation at 10,000 rpm for 10 min, and washed twice with DI water.

To prepare the HAZ NPs, the coordination effect between HA and Zn^2+^ was used to modify the ZIF NPs [29]. The 2 mg/mL of ZIF or ZIF@Fu NPs solution was mixed with 1 mg/mL of HA solution at the same volume under ultrasonic. Stirring for 1 h at 1000 rpm, the obtained precipitate was washed with DI water twice for further use. All NPs were coated with PVP unless otherwise noted. After centrifugation of the ZIF, HAZ, ZIF@Fu and HAZ@Fu prepared from a 20 mL 2-MIM system, the resulting precipitates were freeze-dried, and the powders were directly weighed to determine the yield of NPs corresponding to the volume of the reaction system.

The Fu-loading ratio of HAZ@Fu and ZIF@Fu were detected by an indirect method [48]. The total weight of the ZIF, HAZ, ZIF@Fu and HAZ@Fu (fabricated with 20 mL 2-MIM aqueous solution and 4 mL Zn(NO_3_)_2_ aqueous solution) was measured after freeze-drying, then the ZIF@Fu or HAZ@Fu was destroied by 10 mL ethylene diamine tetraacetic acid (EDTA, 0.1 M), utilizing a semipermeable membrane with a 1000 MWCO to retains loaded Fu and remove other molecule include Zn^2+^, 2-MIM and so on, then the remaining solution was then lyophilized and weighed to obtain the mass of the encapsulated Fu, then the remaining solution was then lyophilized and weighed to obtain the mass of the encapsulated Fu. The drug-loading effeciency was calculated according to: Drug loading (%) = W_drug_/W_total_ × 100%.

We also assessed the sustained release properties of the HAZ@Fu nanoparticles by quantifying the zinc ions by a Zinc Colorimetric Assay [49]. Dissolve 10 mg of HAZ@Fu precipitate thoroughly using hydrochloric acid (0.1 mM) and measure the total zinc ion concentration in the dissolution system. Next, place 10 mg of HAZ@Fu in a centrifuge tube, and resuspend it in 5 mL of phosphate-buffered saline (PBS) at pH 6.0 and pH 7.4, respectively. After reaching each time point, centrifuge the system, withdraw the supernatant for zinc ion concentration analysis, promptly replenish the system volume, and repeat this operation at the next time point to determine the Zinc release profile from HAZ@Fu.

### Antibactirial activity of Fu, MOF NPs and Fu-loaded MOF NPs

The antibactirail activity and MBCs of Fu and Fu-loading NPs were determined for axenic planktonic populations of MRSA using the CFU assay [51]. 100 µL double strength LB broth was dispensed in EP tubes, added the stationary phase cultures of MRSA grown in LB broth were adjusted to density of 2 × 10^6^ CFU/mL. Solutions of free crude Fu, Fu, ZIF were prepared in sterile PBS and added into the EP tubes. All samples were incubated and shaken at 37 °C for 24 h. Then 100-fold dilutions of aliquots (100 µL) from every tube were plated onto sterile LB agar. After incubation at 37 °C, the MBC endpoint was determined as the lowest concentration that resulted in no visual growth of colonies after 24 h of incubation. Each experiment was performed in triplicate (*n* = 3).

The ZOI detection was conducted to test the antiplanktonic bacteria ability. The agar plates were incolated by 100 µL MRSA suspension (1 × 10^6^ CFU/mL), filter papers with a diameter of 6 mm (added 30 µL agents at different concentration) or MNs were put on plates, incubated at 37℃ for 24 h, the digital images of plates were obtained and diameters of ZOI were analyzed.

### Synthesis and characterization of NPs-loaded MNs

The MOF NPs or Vancomycin (Van)-loaded MNs were manufactured through a two-step casting process [47, 56]. 1 g of GelMA was dissolved in 10 ml of PBS solution at 50 °C until fully dissolved. 25 mg Photoinitiator LAP were added to the GelMA solution under ultrasonic. Different NPs (0.90 mg/mL) or Van (60 µg/mL) mixed with above GelMa-LAP solution was used to make the tip of MNs, and PVA solution (w/v = 20%) was made the dissoluble backing layer of MNs. 100 µL tip solution (contained 90 µg NPs or 6 µg Van) added to the MNs mold and placed in a vacuum environment at 50 °C remove air bubbles at the bottom, then the mold were exposed to UV light for gel. Then, 300 µL 20% PVA solution was added to completely cover the tips, dried at 37 °C for over 12 h. Finally, the MNs were detached from the mold for further use.

### Mechanical strength test

The MNs were put on a horizontal surface of an electronic tension tester (Instron 5944) with their needle tips facing upwards. The force sensor approached the MNs at the speed of 0.5 mm/s, the compression force was recorded as long as the mechanical sensor touched the needles and ended when reaching the measured value of 100 N.

### In vitro cell experiments

#### Cytotoxicity test

The biocompatibility of NPs and Fu on RAW 264.7 cells in vitro were evaluated by standard CCK-8 assays, cell viability (%) = (ODsample - ODblank) / (Odcontrol –ODblank) × 100%. The sample group was treated with different solution, while the control group was treated with culture medium.

#### The cellular uptake of ZIF and HAZ NPs

The cell uptake effciency of NPs were detected by CLSM and FCM. 1.5 mL of RAW 264.7 (200,000 cells/well) were seeded in dishes and incubated for 24 h (with or without 100 ng/mL LPS). Cells were washed three times with PBS, treated with 1.5 mL DMEM containing HAZ@FITC or ZIF@FITC (60 µg/mL) for specific time point. Then, medium was removed and cells were washed three times with PBS. For CLSM, cells were stained with DAPI under 37℃ for 15 min after fixation, images were immedately recorded. For FCM, after incubated with FITC-loaded NPs for 1 and 3 h, cell suspensions were centrifuged for 6 min at 1500 rpm at 4 °C. Supernatants were removed and RAW 264.7 pellets were stained with APC anti-mouse F4/80 antibody (Biolegend, USA), FITC and APC fluorescence of each well was detected by FCM.

#### RNA sequencing analysis

A microarray was used to detect the gene expression profle of macrophages on samples. Briefly, BMMs were seeded (1 × 10^6^ per well) and cultured with or without HAZ@Fu for 24 h. Then, cell total RNA was harvested by Trizol reagent, and the whole gene expression was examined at the Guangzhou Genedenovo Technology Service Co., Ltd. (China). Fold changes in the expression of genes were exhibited by a heat map, and pathway enrichment was evaluated by GO, Reactome and KEGG pathway analysis.

#### Intracellular fate of ZIF and HAZ NPs

1 mL RAW 264.7 (10,000 cells/well) were seeded in a confocal dish and incubated for 24 h. Cell monolayers were then washed with PBS and incubated with 60 µg/mL ZIF@FITC or HAZ@FITC in 1 mL complete DMEM for 4 h. The cells received DMEM as the negetive control group. The dish was washed three times with PBS and incubated with 500 µL of Lyso-Tracker Green DND-99 staining in DMEM, for 5 min at 37 °C. Images were immediately recorded. PCC [57] was used to quantify the degree of colocalization between fluorescent FITC and lysosomes or intracellular S. aureus. PCC was calculated using the Image J software. We analysed a minimum of 6 microscopic vision fields randomly taken from each slide.

#### Cell infection and subcellular targeting ability of HAZ NPs

For investigate whether HAZ NPs provide a subcellular targeting to intracellular MRSA [36], 1 mL RAW 264.7 (30,000 cells/well) were seeded in a confocal dish and incubated for 24 h, removed PBS and added 1mL mid-log phase MRSA (USA300 transferred by meCherry, 3 × 10^6^ CFU/mL) containd pen/strep free DMEM medium, incubated for 1 h at 37℃. Then we added FBS and pen/strep free DMEM containing 2×MIC Van (5 µg/mL) and incubate for 1 h to remove any extracellular bacteria, then remove medium and washed twice with PBS. Infected cells were treated with HAZ@FITC contained DMEM (60 µg/mL) for 3 h, then washed with PBS for 3 times, recorded by CLSM.

#### Evaluation of intracellular antibacterial activity

According to previous research [33], RAW 264.7 cells (1.5 × 10^5^/well) in 1 mL complete DMEM was seed onto 24 well plates and incubated for 24 h, Mid-log phase MRSA (ATCC 43,300) were washed twice and adjusted to 7.5 × 10^6^ CFU/mL in pen/strep free DMEM to give a cell 500 multiplicity of infection. Treated with MRSA for 1 h, 2×MIC Van (5 µg/mL) and incubate for 1 h to remove any extracellular bacteria. Then the cell were incubated with Fu, ZIF NPs, HAZ NPs, ZIF@Fu NPs and HAZ@Fu NPs (all groups at an equivalent concentration of 60 µg/mL) for 2 or 5 h, respectively. Then the cells were lysed with 0.025% Triton X, and lysates were harvested and serially diluted 100-fold with PBS, and then 100 µL of diluted solution was inoculated onto LB agar plates, incubated for 24 h, then digital images of plates were obtained and viable counts were performed.

### In vivo wound healing evaluation

Female BALB/c mice were used to establish a diabetic wound model. Strain of MRSA (ATCC 43,300) was inoculated in LB borth and shaken for 16 h in 37 °C. Then, the bacteria were washed twice and resuspended with PBS and diluted to 0.75 × 10^8^ CFU/mL. An air isoflurane anesthesia machine was used to anesthetize the mice, after skin preparation, surgery was performed on the back of each mouse to make a circular wound with a diameter of 8 mm. Then 50 µL MRSA contained PBS (0.75 × 10^7^ CFU/mL) was seeded onto the wound. After 24 h, all mice were randomly divided into 6 groups (control group, ZIF@Fu MNs group, HAZ@Fu MNs group, ZIF MNs group, HAZ MNs group and Van MNs group, *n* = 4), the mice were anesthesia and MNs were applied directly to the wounds with appropriate pressure to enable the microneedles to penetrate the skin. The patches were then further secured with adhesive skin dressings. After 8 h, the dressings were removed without any additional cleansing of the wounds. Each mouse was reared in a single cage with water and food. The mice in each group were photographed at 0, 4, 6, 8, 10 days after the wound treatment, and the wound area was calculated using Image J software. The wound area rate was measured using the following formula:

Wound area (%) = Actual wound area / Wound area at day 0.

Another 18 mice infectious wound model were estabished, and divided into 6 groups (*n* = 3), treated as mentioned above. On the day 6, we harvested the skin tissue which containing the whole wound area of all groups, immerse in 1 mL PBS for tissue homogenization, the homogenate was serially diluted 100-fold with PBS, and then 100 µL of diluted solution was inoculated onto LB agar plates, digital images of plates were obtained and viable counts were performed.

### Histology and immunohistochemistry staining

After 10 days of treatment, all mice were sacrificed and harvested the skin tissue containing the whole wound area, further soaked in 4% paraformaldehyde for 24 h. The samples were embedded in wax blocks and then prepared into 5-µm-thick sections for histological staining. The antibacterial effciency of every group was evaluated by Giemsa staining. The formation of granulation tissue and collagen during wound healing was detected by using hematoxylin and eosin (H&E) and Masson trichrome staining. The tissue inflamation and regeneration were dectected by immunohistochemical staining of CD206 (M2 maker) and IL-6 (inflammatory factor). For neovascularization analysis, antibodies of CD31 and α-SMA were utilized to stain endothelial cells and fibroblasts, respectively.

### Statistical analysis

Results were obtained from three or more independent experiments. Data analysis was conducted using GraphPad Prism 8 software (GraphPad Software, San Diego, CA, USA). In each group, at least three technical replicates were set up, and the results were presented in the form of mean ± standard deviation (SD). Differences between groups were analyzed by one-way analysis-of-variance (ANOVA), Asterisks denote statistically significant differences (*p < 0.05, **p < 0.01, ***p < 0.001, and ****p < 0.0001, ns: not significant).

### Electronic supplementary material

Below is the link to the electronic supplementary material.

Supplementary Material 1

## Data Availability

All data generated and analyzed during this research are included in this published article.
