# Supplementary material for: Multifunctional fucoidan-loaded Zn-MOF-encapsulated microneedles for MRSA-infected wound healing
Source: J Nanobiotechnology. 2024 Apr 4;22:152. doi: 10.1186/s12951-024-02398-4 (PMC10996189; doi:10.1186/s12951-024-02398-4)
Supplement: Supplementary file 1 — Supplementary Material 1 [file 12951_2024_2398_MOESM1_ESM.docx]

**Mutifunctional Fucoidan-loaded Zn-MOF Encapsulated Microneedles for MRSA** **Infected Wound Healing**

**Zichao Jiang^1,3,4^, Jingyi Li^1,3,4^, Jiahao Wang^1,3,4^, Yixiao Pan^1,3,4^, Shuailong Liang^1,3,4^, Yihe Hu^2,4^**†, **Long Wang^1,3,4,5^**†

^1^Department of Orthopedics, Xiangya Hospital, Central South University, China, ^2^Department of Orthopedics, First Affiliated Hospital, School of Medicine, Zhejiang, China,

^3^University Hunan Engineering Research Center of Biomedical Metal and Ceramic Implants, Xiangya Hospital, Central South University, China,

^4^National Clinical Research Center for Geriatric Disorders, Xiangya Hospital, Central South University, Changsha, China,

^5^Hunan key laboratory of aging biology, Xiangya Hospital, Central South University, Changsha, China,

†: Corresponding to Long Wang, MD, Ph.D., E-mail: [dr_wanglong@csu.edu.cn](mailto:dr_wanglong@csu.edu.cn) and Yihe Hu, MD, Ph.D. E-mail: [huyh1964@163.com](mailto:huyh1964@163.com)

**Table. S1.** List of Abbreviations in Order of Appearance.

| Microneedle patches | MNs |
| --- | --- |
| metal-organic framework | MOF |
| zeolitic imidazolate framework-8 | ZIF-8 |
| low molecular weight fucoidan | Fu |
| hyaluronic acid | HA |
| *Staphylococcus aureus* | *S. aureus* |
| Methicillin-resistant *Staphylococcus aureus* | MRSA |
| gelatin methacryloyl | GelMA |
| polyvinyl acetate | PVA |
| Kyoto Encyclopedia of Genes and Genomes | KEGG |
| minimum inhibitory concentration | MIC |
| vancomycin | Van |
| Cluster of differentiation 44 | CD44 |
| 2-methylimidazole | 2-MIM |
| scanning electron microscope | SEM |
| dynamic light scattering | DLS |
| polydispersity index | PDI |
| Transmission Electron Microscopy | TEM |
| X-ray diffractometer | XRD |
| Fourier transform infrared spectroscopy | FTIR |
| colony-forming unit | CFU |
| minimum bactericidal concentrations | MBCs |
| Luria-Bertani | LB |
| zones of inhibition | ZOI |
| polydimethylsiloxane | PDMS |
| flow cytometry | FCM |
| Lipopolysaccharide | LPS |
| Bone marrow-derived macrophages | BMMs |
| differentially expressed genes | DEGs |
| Gene Ontology | GO |
| biological processes | BP |
| kyoto encyclopedia of genes and genomes | KEGG |
| confocal laser scanning microscopy | CLSM |
| Pearson’s correlation coefficient | PCC |
| green fluorescent protein | GFP |
| Hematoxylin and eosin | H&E |
| α-smooth muscle actin | α-SMA |
| Polyvinyl pyrrolidone | PVP |
| Lithium Phenyl ( 2,4,6-trimethylbenzoyl ) phosphinate | LAP |


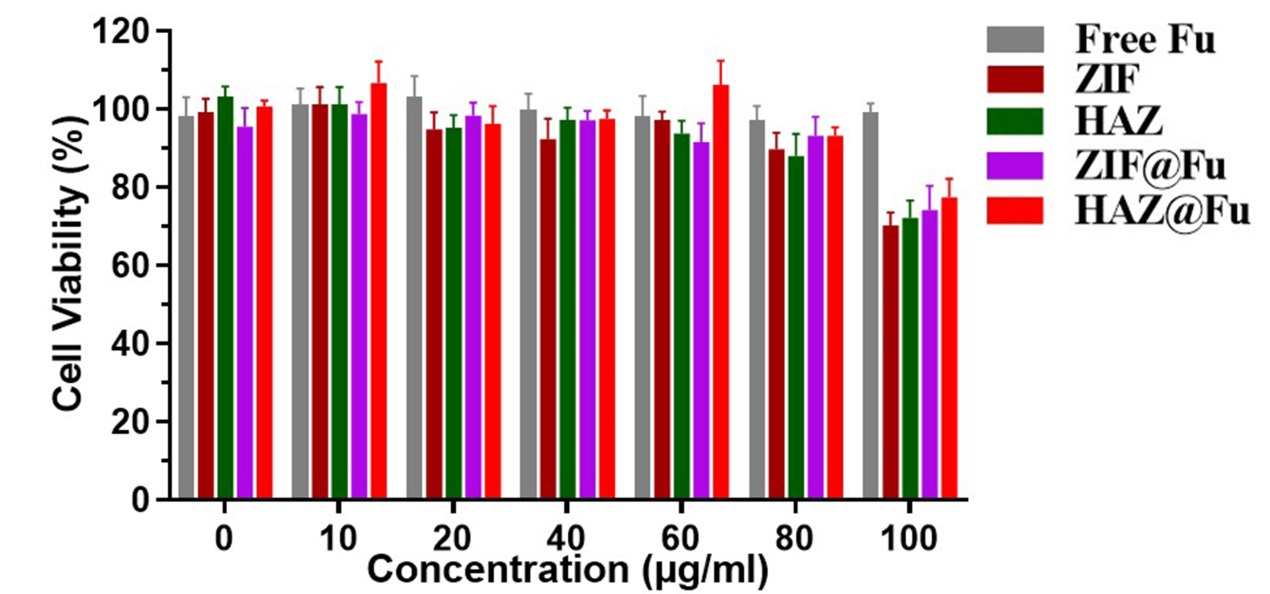


**Fig. S1.** Cell viability of RAW 264.7 treated with various concentrations of Fu and Fu-loading NPs for 24 h.


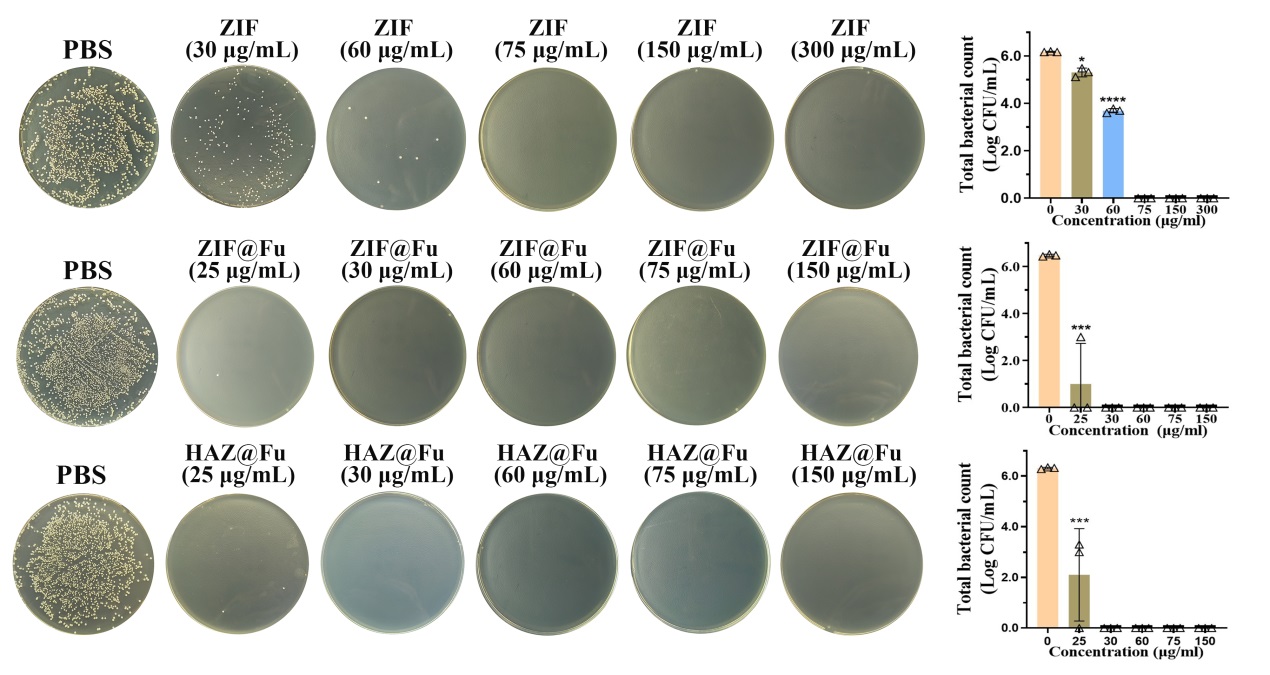


**Fig. S2.** *In vitro* MRSA strains (1×10^6^ CFU/mL) resuspend in PBS incubated with ZIF, ZIF@Fu and HAZ@Fu for 16 h, then serially diluted (100×) in sterile PBS and inoculated onto LB agar plates and incubated for 24 h.


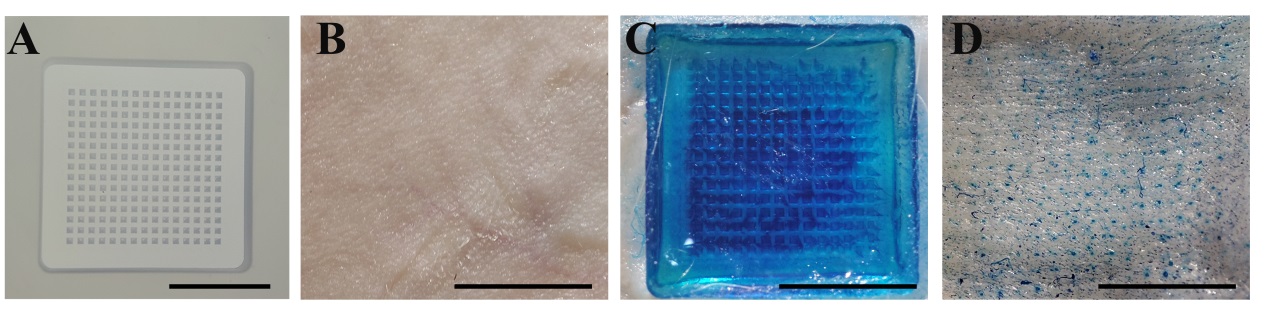


**Fig. S3.** Photograph of A) Photographic image of the PDMS MN mold, B) mice back skin, C) methylene blue-stained GelMA-MNs (10% w/v) applied to mice back skin. (D) The traces left after removing the applied MNs. Scale bar: 5 mm


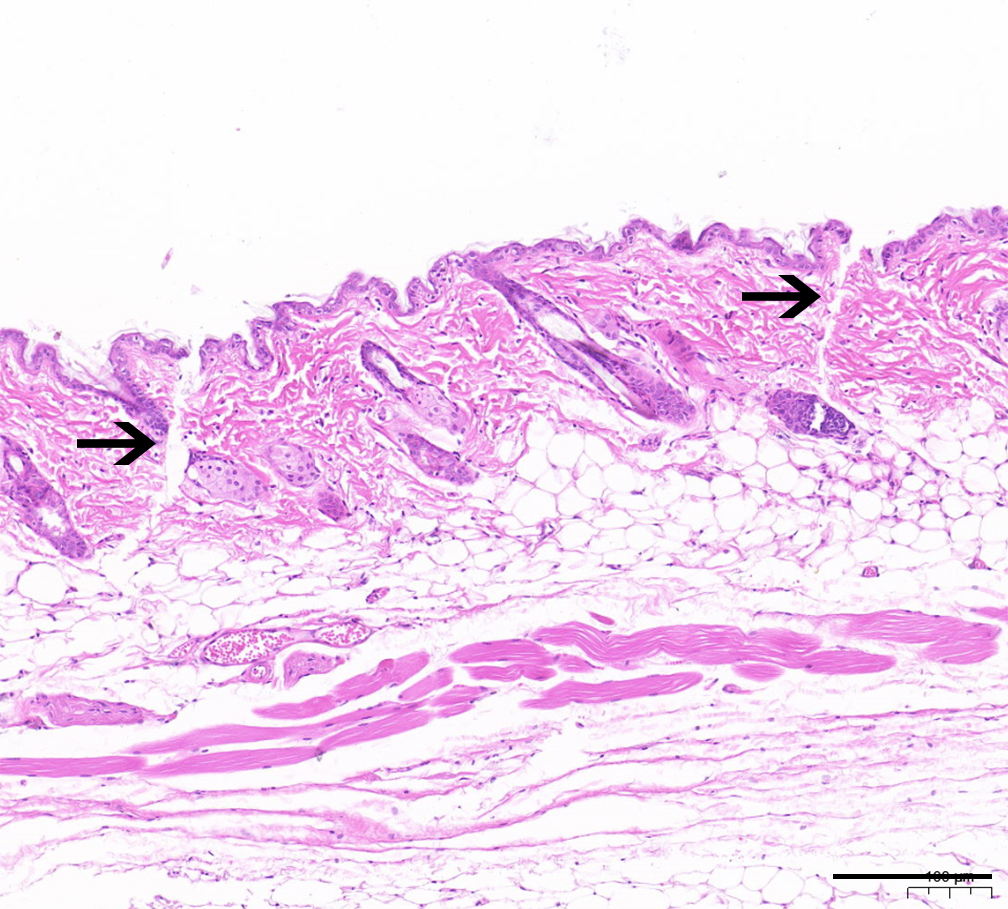


**Fig. S4.** Skin insertion capability of HAZ@Fu MN. Histological frozen section after insertion into mouse skin and stained with H&E.


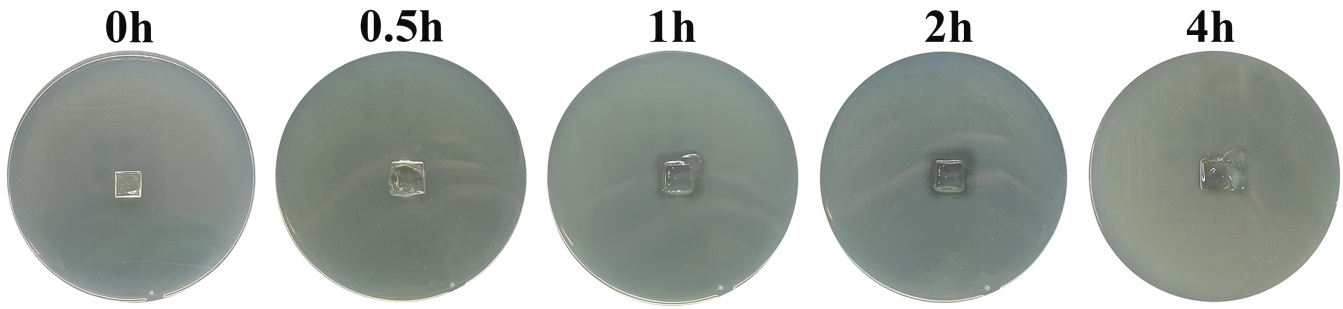


**Fig. S5.** Dissolution process of HAZ@Fu MN after inserting into the LB agar plate at 37°C.


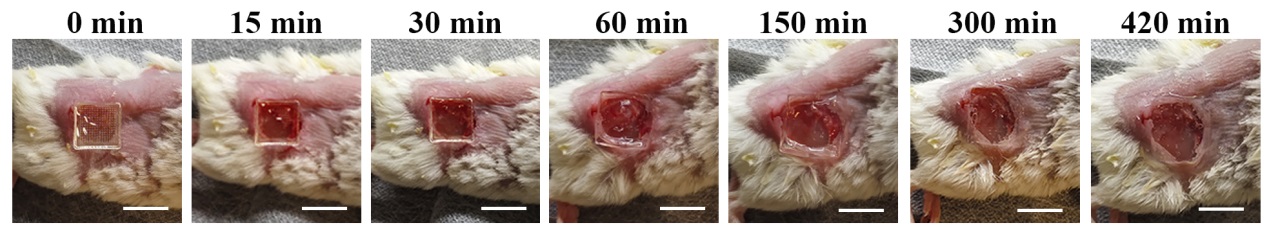


**Fig. S6.** The dissolution process of HAZ@Fu MN after administrated to skin wound of mice (Scale bar: 1 cm).


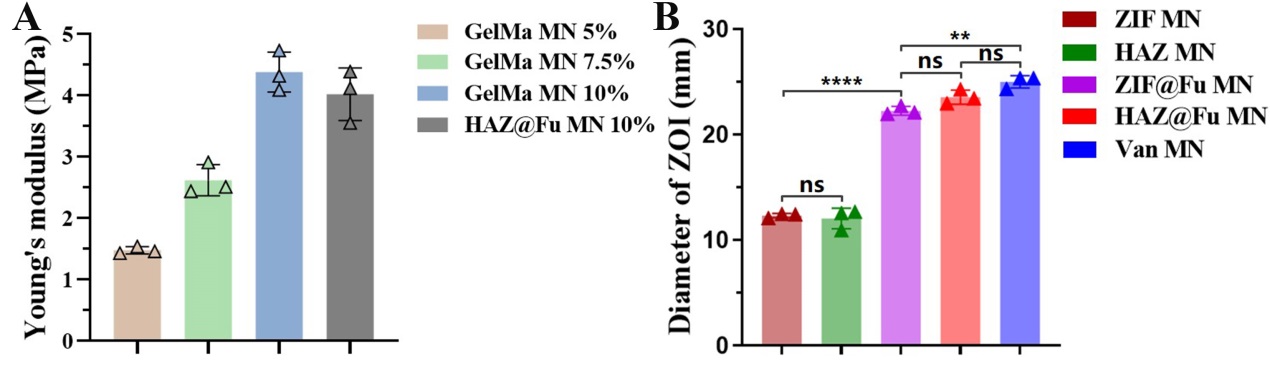


**Fig. S7**. A) Young’s modulus of MN patches with different compositions. B) Result of ZOI diameters of ZIF MNs, HAZ MNs, ZIF@Fu MNs, HAZ@Fu MNs and Van MNs against MRSA.


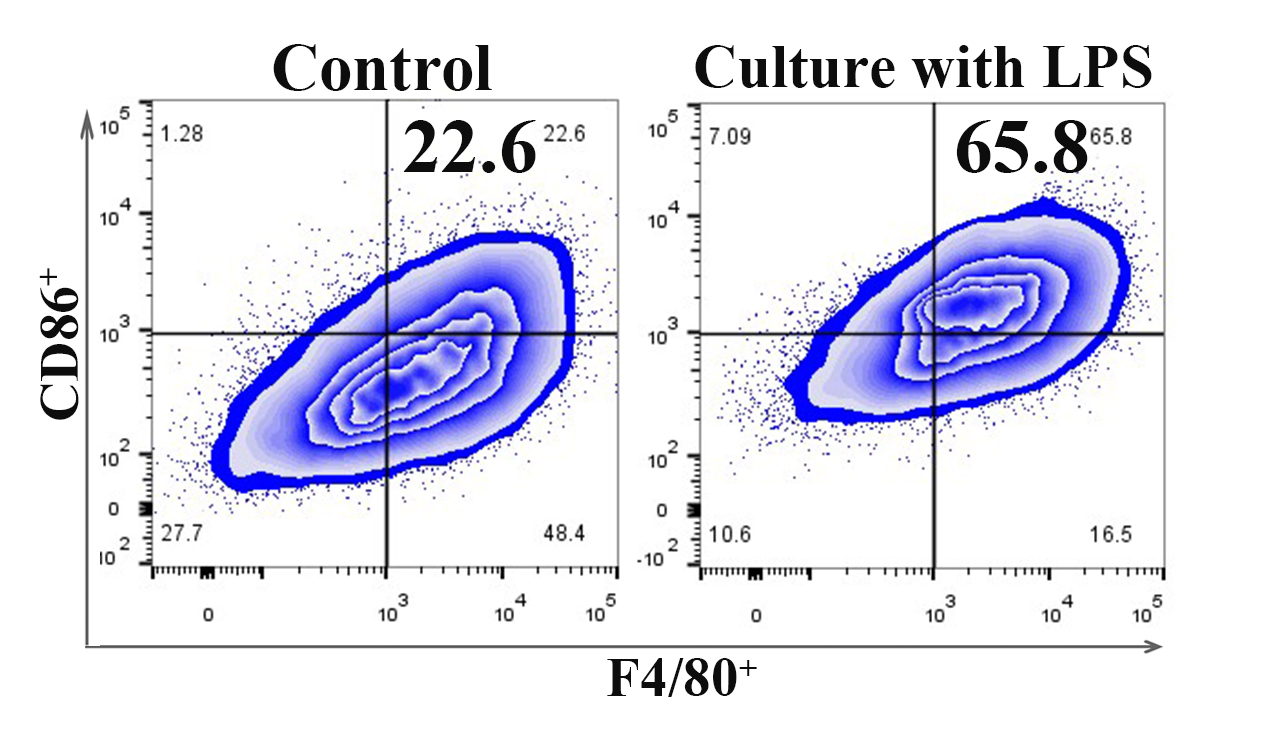


**Fig. S8.** FCM results of RAW 264.7 cells cultured with normal medium and medium with LPS (100 ng/mL) after 24 h, stained with M1 maker CD86.


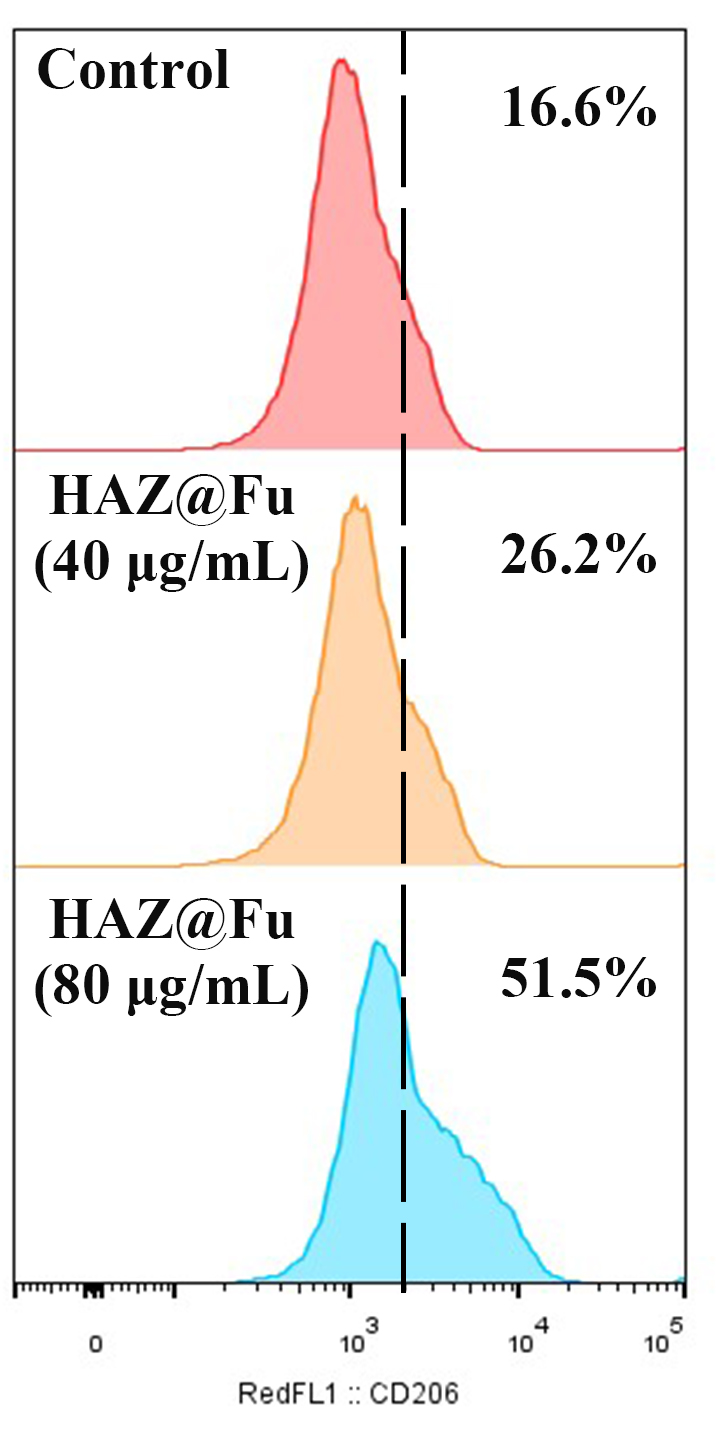


**Fig. S9.** FCM results of BMMs co-cultured with normal medium and medium with HAZ@Fu NPs after 48 h, stained with M2 maker CD206 (gated by F4/80).


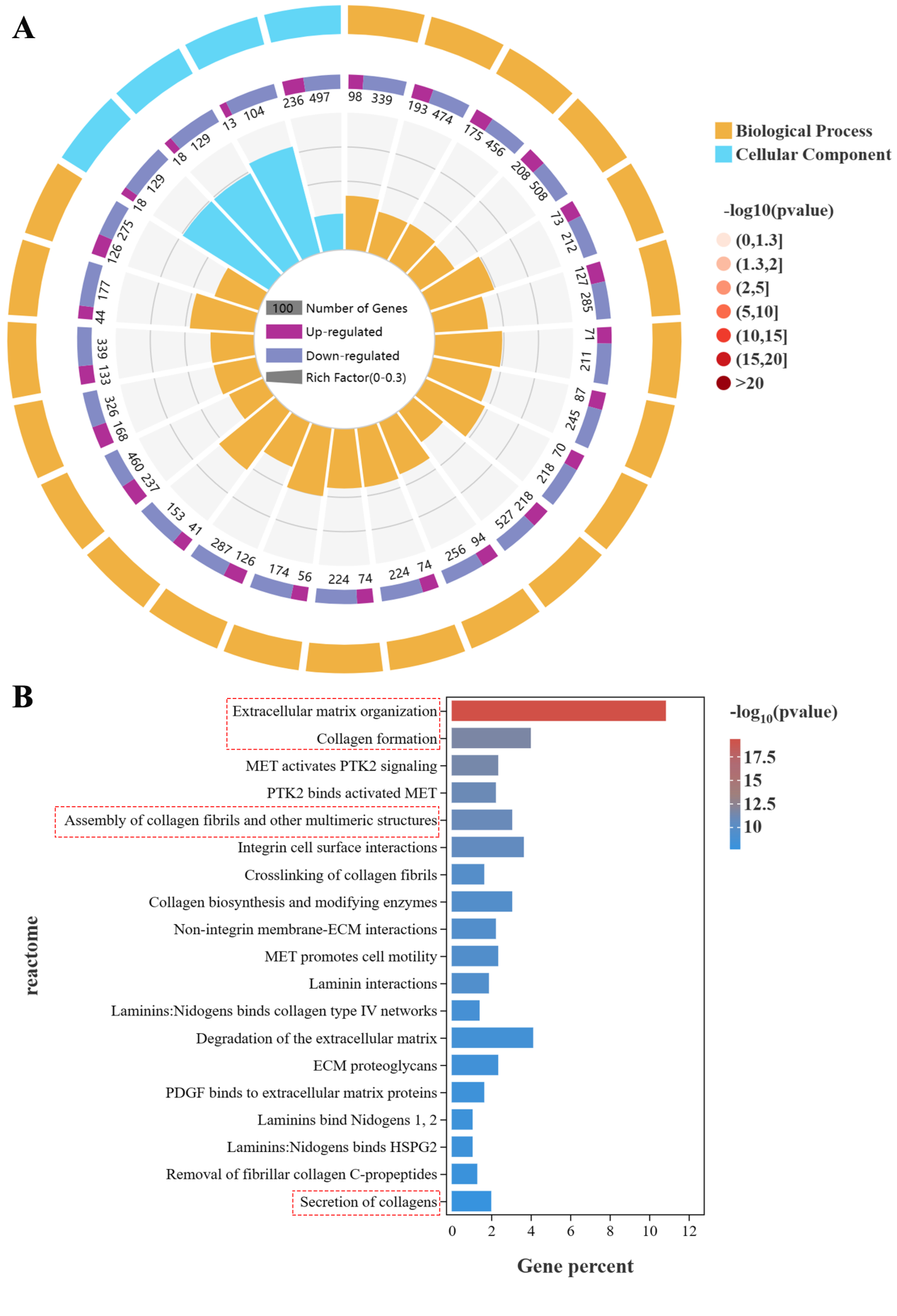


**Fig. S10.** A) Circle plot through GO analysis to visualize the top 25 DEGs. B) Representative pathways analyzed by Reactome pathway method.


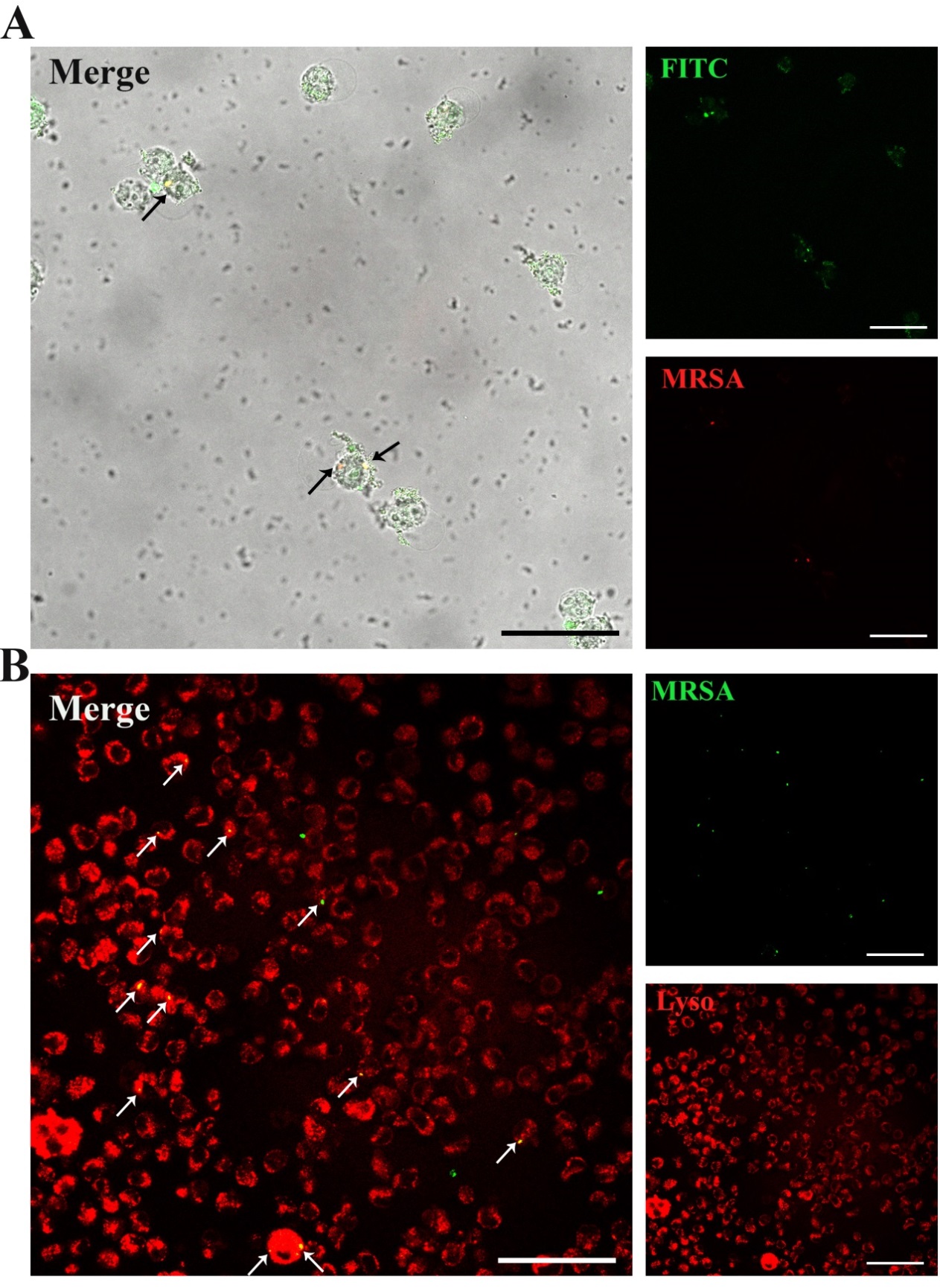


**Fig. S11.** A) HAZ NPs colocalization with intracellular MRSA, RAW 264.7 were incubated with MRSA (transferred by GFP), removed extracellular bacteria and washed with PBS, further incubated with 80 μg/mL HAZ@FITC NPs for 3 h, washed twice for confocal laser scanning microscope (scale bar: 50 μm). B) RAW 264.7 cells were infected by MRSA (transferred by GFP) and removed extracellular bacteria. Slides were washed with PBS and treated with Lyso-Tracker Red for 5 min at 37 °C. Images were immediately recorded on living cells (scale bar: 50 μm).


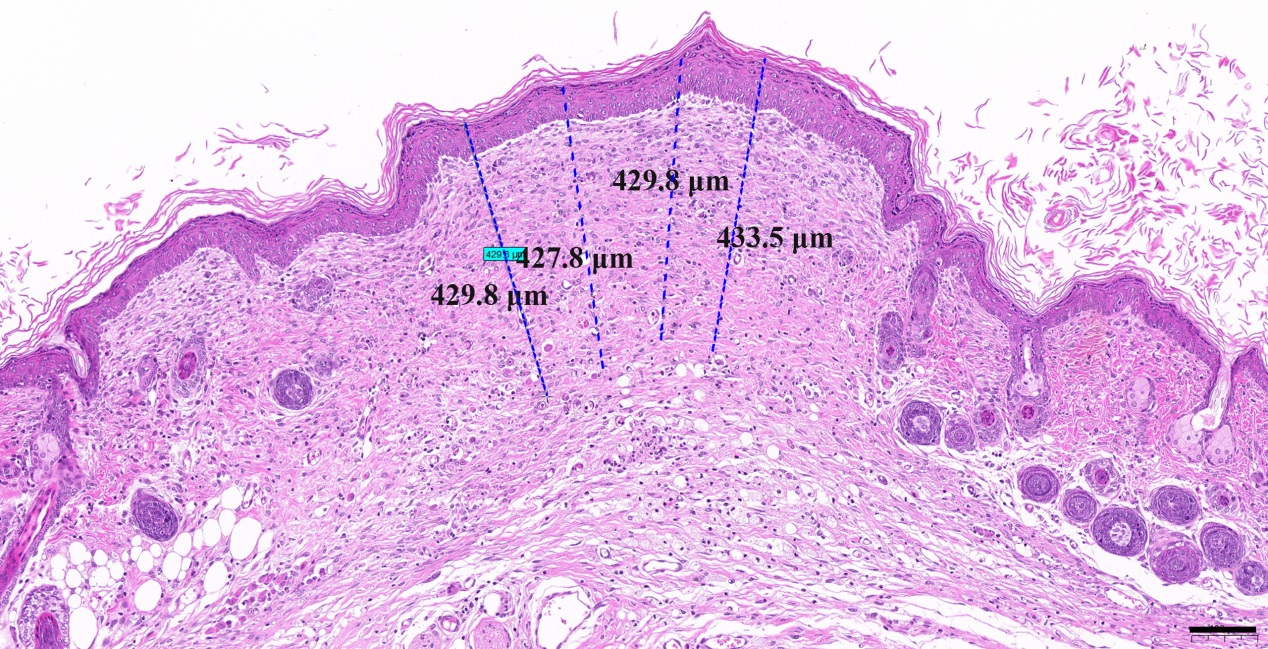


**Fig. S12.** Example of detection of granulation tissues in HAZ@Fu MN groups, randomly select three to four positions to measure and take the average value for every sample (scale bar: 100 μm).
